# Mode of Action of Diterpene and Characterization of Related Metabolites from the Soft Coral, *Xenia elongata*

**DOI:** 10.3390/md12021102

**Published:** 2014-02-20

**Authors:** Eric H. Andrianasolo, Liti Haramaty, Eileen White, Richard Lutz, Paul Falkowski

**Affiliations:** 1 Center for Marine Biotechnology, Institute of Marine and Coastal Sciences, Rutgers, The State University of New Jersey, New Brunswick, NJ 08901, USA; E-Mails: andriane@marine.rutgers.edu (E.H.A.); haramaty@marine.rutgers.edu (L.H.); rlutz@marine.rutgers.edu (R.L.); 2 The Cancer Institute of New Jersey, 195 Little Albany Street, New Brunswick, NJ 08903, USA; E-Mail: epwhite@cinj.rutgers.edu

**Keywords:** structural and functional characterization of marine drug, marine biotechnology, bioactive compounds and bioproducts, drug discovery and development

## Abstract

Chemical and biological investigation of the cultured marine soft coral *Xenia elongata* led to the isolation of two new diterpenes (**2**, **3**). Their structures were elucidated using a combination of NMR and mass spectrometry. Biological evaluations and assessments were determined using the specific apoptosis induction assay based on genetically engineered mammalian cell line D3 deficient in Bak and Bax and derived from a mouse epithelial cell. The diterpenes induce apoptosis in low micromolar concentrations. The results indicate that the previously isolated compound (**1**) affects cell in a manner similar to that of HSP90 and HDAC inhibitors and in a manner opposite of PI3 kinase/mTOR inhibitors. Compound (**3**) inhibits selectively HDAC6 in high micromolar concentrations.

## 1. Introduction

**Figure 1:**
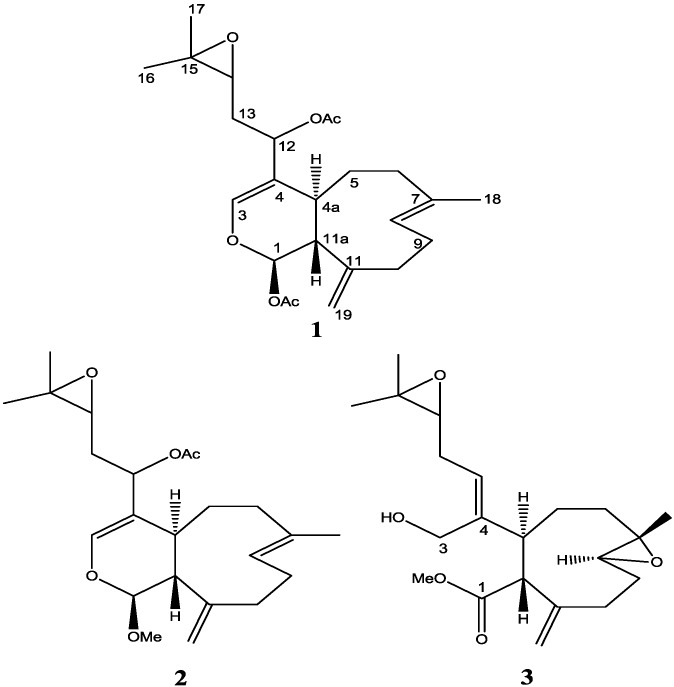
Structure of compounds **1**, **2** and **3**.

Natural products are the most reliable and rich source of new anticancer entities. Over the past 30 years, nearly 63% of anticancer drugs introduced to the pharmaceutical development market are natural products or can be traced from a natural products origin [1,2]. The soft coral of the genus *Xenia elongata* has produced interesting biological molecules, specifically apoptosis inducer compounds [3,4]. These molecules embed a framework of nine-membered rings and a particular arrangement of functional groups with multiple stereocenters. These structural features limit the range of chemical reactions that are applicable to their synthesis. There is little in the existing synthetic literature to define an effective strategy for the synthesis of these molecules [5]. The first total synthesis of an optically active xenicane diterpene has not yet been achieved [5,6]. In our ongoing effort to discover and develop new marine natural product biomedicinals, and in order to explore the diversity of natural products from the soft coral *Xenia elongata*, chemical and biological investigation of this organism were undertaken. Two novel diterpenes (**2**, **3**) were isolated and, remarkably, these new diterpenes induce apoptosis and, more importantly, **3** is 10-fold more active than the most active and previously isolated compound **1** (Figure 1). The detail mode of action of **1** by analysis of MCF-7 breast cancer treated with **1** was also reported here. HDAC inhibitory assay of **3** was also displayed and discussed.

## 2. Results and Discussion

Live colonies of *Xenia elongata* were grown in the coral laboratory at the Marine Biotechnology Center, Institute of Marine and Coastal Sciences, Rutgers University. For extraction, 200 g of the colonies were isolated and frozen. A methanol (or dichloromethane) soluble fraction was extracted from frozen coral tissue, lyophilized and dissolved in DMSO. The extract that induced apoptosis was subsequently fractionated and purified by analytical RPHPLC. Using this strategy, compounds with proapoptotic activity were isolated from the whole tissue extracts. Chemical structures of the two compounds (**2** and **3**) were ascertained by standard spectroscopic techniques as described below.

The molecular formula of **2** was established as C_23_H_34_O_5_ on the basis of HRESIMS (*m*/*z* 413.2299 [M + Na]^+^ calcd. for C_23_H_34_O_5_Na, 413.2298). This indicated a difference of 28 mass units and probably a carbonyl group, compared to compound **1** [3]. The ^1^H NMR (Supplementary Figure S1) of **2** indicated clearly the existence of the following functional groups: an 1-methoxydihydropyran moiety [δ 5.35 (d, *J* = 1.4 Hz), H-1 and 6.51 (s), H-3], a terminal methylene [δ 5.05 (s) and 5.15 (s), H-19, H-19′], two methyls α to oxygen [δ 1.33 (s), H-16, H-17], an epoxy signal at [δ 2.75 (dd, *J* = 5.9, 6.2 Hz), H-14] and a vinyl methyl group [1.60 (s), H-18]. The NMR spectra of compounds **1** and **2** were compared in CDCl_3_ and were found to be similar, with the exception of the carbon at C-1 [δ 99.3] (Supplementary Figure S2) for **2** instead of C-1 [δ 91.7] for **1** in addition to that **2** has one less carbonyl signal, suggesting that a methoxy group is attached to C-1 in **2** instead of acetate group in **1**. The deshielding effect of the methoxy group to the carbon at C-1 position is more pronounced compared to the deshielding effect of an acetate group to the carbon at the same position.

**Figure 2:**
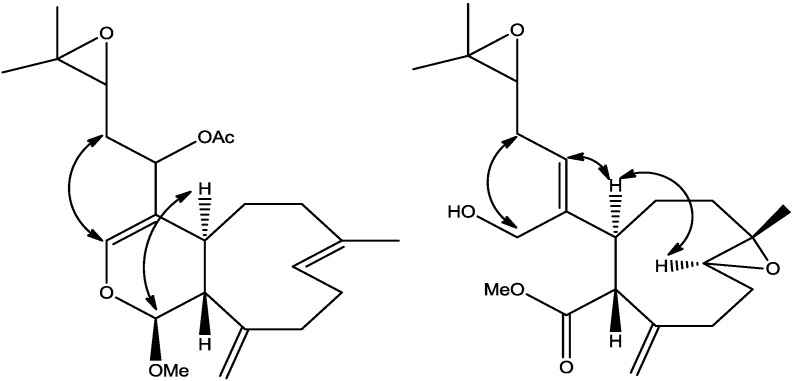
Selected ROESY cross peaks for compounds (**2**) and (**3**).

**Scheme 1:**
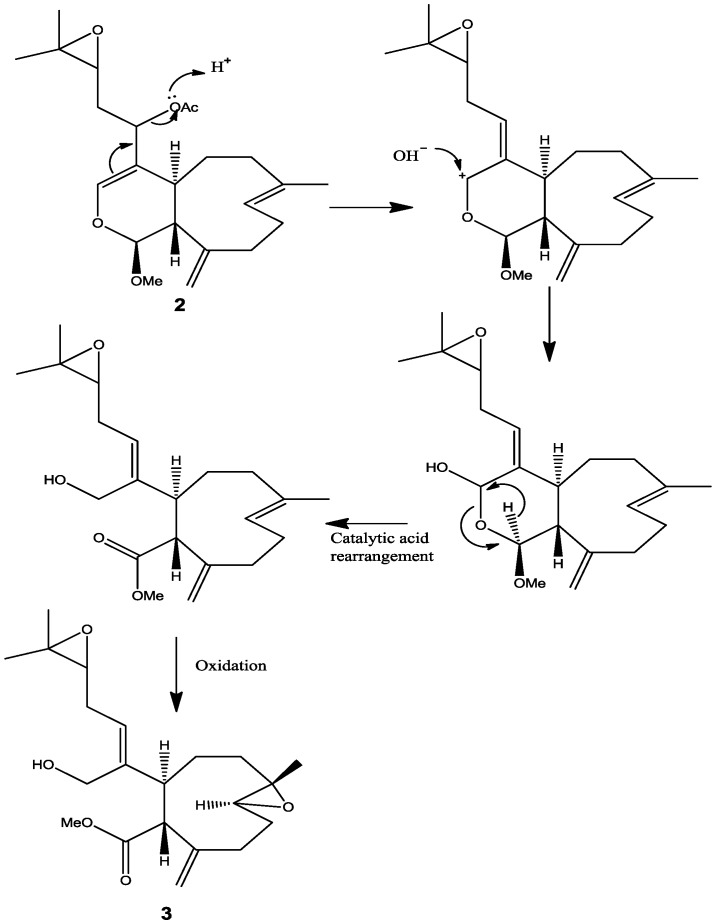
Proposed rearrangement of **2** under acidic conditions, to give **3**.

The relative stereochemistry of **2** was established with the same method as described in our previous work [3] which is based on ROESY data (Supplementary Figure S3), coupling constant analyses and chemical shifts comparison to **1** and xeninculin [7], tsitsixenicin A [8,9] and related compounds [7,9]. The coupling constant (*J* = 12 Hz) between H-4a and H-11a suggests a trans ring junction. The small coupling constant (*J* = 1.4 Hz) between H-1 and H-11a would favor the α*-*position for H-1 if the six-member ring was in a quasi-boat conformation. A closer look of the crystal structure of xenicin and essentially the six-member ring, which is also present in **2**, suggests that H-1, if it is in the α*-*position, should display ROESY cross peak correlation to H-4a. In order to verify this suggestion, a high scan ROESY experiment was performed. Careful analysis of the ROESY data, particularly in the cross region of H-1 and H-4a, revealed unambiguously that these two protons display a ROESY cross peak correlation (Figure 2). Based on this result, it appears that the configurations at C-1, C-4a, and C-11a in compound **2** are essentially the same as those in **1** and xeninculin, in which the protons H-1 and H-4a are on the α face of the ring and the proton H-11a is oriented on the β*-*side. This observation confirmed that the stereochemistry of this proton at H-1 position is well conserved and common to any molecule with dihydropyran moiety in the xenicane diterpene class. Previous attempts to establish the stereochemistry of an acetate group at C-12 in xenicane diterpenes by chemical transformations and spectroscopy have failed [9,10], and therefore the stereochemistry at C-12 remains unassigned. This information suggested that the acetate group attached to C-12 is very reactive and probably leads to further transformation of the molecule as described by the proposed rearrangement in Scheme 1. From the above results, the structure of **2** was formulated as shown.

A molecular formula of C_21_H_32_O_5_ for **3** was determined from HRESIMS data (Supplementary Figure S4 Low resolution). ^1^H NMR (Supplementary Figure S5) of **3** indicated the presence of two epoxy signals at [δ 2.80 (dd, *J* = 11.5, 3.2 Hz), H-8] and [δ 2.76 (dd, *J* = 7.2, 6.1 Hz), H-14], one methyl α to oxygen [δ 1.16 (s), H-18] and a terminal methylene [δ 5.00 (s) and 5.20 (s), H-19, H-19′]. The presence of two equivalent protons at δ [3.72, d(6)], H-3 for an isolated methylene attached to oxygen indicated that **3** has a primary alcohol functional group at C-3 which also revealed that the dihydropyran moiety presents in **1** and **2** is absent in **3**. The NMR data of **3** were analogous to those of diterpene xenitacin isolated from the Formosan soft coral *Xenia umbellata* [4] except that xenitacin had an acetate group attached to C-3 instead of a hydroxy group for **3** at C-3 (Supplementary Figure S6).

**Figure 3:**
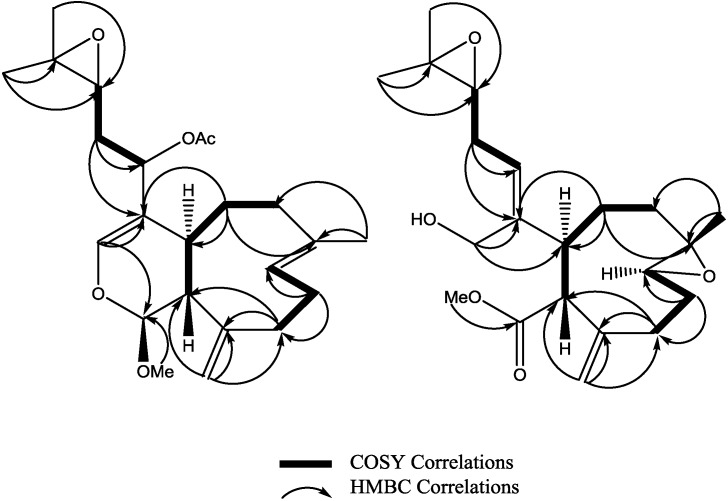
Key HMBC and selected COSY correlations for compounds (**2**) and (**3**).

The relative stereochemistry of **3** was investigated with the aid of a ROESY spectrum. The *Z*-configuration was assigned to Δ^4(12)^ double bond based on the observed ROESY cross peak between H-12 [δ 5.45] and H-4a [δ 3.29]. The configuration of the two chiral centers at C-4a and C-11a was deduced by ROESY data and comparison with those of xenitacin [4]. The large coupling constant (*J* = 12 Hz) between H-4a [δ 3.29] and H-11a [δ 3.45] suggests that they have a configuration opposite to each other [4]. The observed ROESY cross peak between H-4a [δ 3.29] and H-8 [δ 2.80] (Figure 2, Supplementary Figure S7) suggests that they have similar configuration to xenitacin [4]. Therefore the structure of **3** was established as shown. Key HMBC and selected COSY correlations were established for **2** and **3** in order to assign all carbons framework in their structures (Figure 3 and Supplementary Figures S8–S13).

In order to assess the pro-apoptotic activities of these compounds as a marker for their potential anticancer efficacy, methanol soluble fractions of *Xenia* extracts were tested for apoptosis induction in an MTT assay using the apoptosis competent W2 and apoptosis resistant D3 cells, as described in our previous work [3]. Compounds **2** and **3** induce measurable cell death in W2 but not in D3 cells (Supplementary Figures S14 and S15). To quantify apoptosis induction, viability was determined using an MTT assay [11]. Measurements were taken at time 0 and 48 h after addition of compounds in different concentrations. Viability was calculated as the difference between time 0 (addition of compound) and 48 h. Apoptosis induction was defined as at least 20% death of W2 cells and a 10% or higher growth of D3 cells by MTT assay. The optimal concentration of the compounds for apoptosis induction was determined based on the dose response of the two cells lines by MTT assay. The results reveal a significant induction of apoptosis by the whole tissue extract and by individual compounds (11.5 µM for **2** and 1.2 µM for **3**), with the most remarkable induction shown by compound **3** (1.2 µM) which is ten times more active than compound **1** previously characterized [3]. Treatment of W2 and D3 cells with 0.1 µM staurosporine, a protein kinase inhibitor and potent inducer of apoptosis, was included as a positive control for apoptosis induction and for comparison. Thus, the diterpenes we have isolated and characterized effectively and specifically activate apoptosis in immortalized mammalian epithelial cells.

There are efforts underway to synthesize optically active xenicane diterpene [12] and work is in progress for the synthesis of diterpene from *Xenia elongata*. One synthetic compound (Supplementary Figure S16) from this work which has the same structure of **3** but does not have the side chain was also tested for comparison of activity. The result revealed that this synthetic compound did not induce apoptosis, which suggested that the side chain is critical for the activity. It is also reported before that a similar structure, xenitacin, isolated from the Formosan soft coral *Xenia umbellata* [4], is the most active compound in its group in a cytotoxicity assay against P-388, HT-29 and A549 cells [4]. These bioactivity results may suggest that a conversion or rearrangement toward the most active structure may undergo within the organism itself or even in the cell in order to increase the efficacy of affecting or killing cancer cells. A proposed conversion or rearrangement of **2** to **3** is displayed (Scheme 1). It is also possible that **1** will convert to **2** by enzymatic reaction of deacetylation followed by methylation and then **2** will be converted to **3**.

In order to determine the mode of action of diterpenes from *Xenia elongata*, a connectivity analysis for MCF-7 breast cancer cells treated with compound **1** was performed. The objective is to analyze changes in gene expression in a breast cancer cell line exposed to a soft coral diterpene, which is compound **1** in this experiment, and to compare the resulting signature to perturbagen signatures in the Connectivity Map [13].

To prepare a 0.12 M stock solution in DMSO, 1.6 mg of **1** was used. Proliferating MCF-7 breast cancer cells were treated in triplicate with either a 120 µM final concentration of **1** (final DMSO concentration 0.1%), or were left untreated for 8 h. Cells were harvested before the RNA was extracted then purified, reverse transcribed, and hybridized to an Affymetrix array by the CINJ Transcriptional Profiling Core. The resulting gene expression data were analyzed.

Expression levels of 22,277 genes in three untreated and three treated samples were obtained using an Affymetrix array. For this initial analysis, the 11,400 genes were used for which all six samples provided clearly measurable signals. (Genes for which any sample was determined to be “A” or “M” were excluded, and only those for which all six were called “P” by the Affymetrix software were kept).

The package “siggenes” [14] was used in the bioconductor set of gene expression packages for the R statistical system. Two different methods were considered for obtaining the most differentially expressed genes. The first method is known as “significance analysis of arrays” or “SAM” and was first proposed by Tusher *et al*. [15]. This method computes a modified t-statistic comparing treated to control samples for each gene. It then identifies differentially expressed genes so that the false discovery rate (FDR) is a pre-specified value. The second method, known as “Empirical Bayes Analysis of Microarrays” or “EBAM” [16] also computes the modified t-statistic. Unlike SAM, however, it computes the posterior probability that each gene is differentially expressed. Each of the two methods can be used to create a list of genes that are differentially expressed. The two lists will generally be different, although there is usually some overlap.

**Figure 4:**
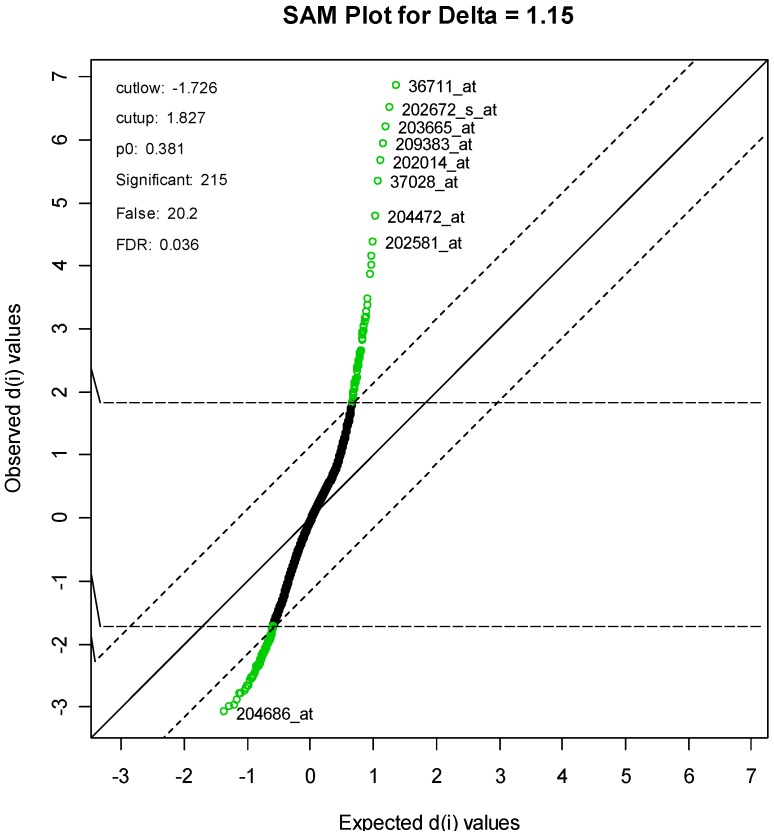
SAM Plot, with some genes labeled.

For the SAM method, the parameter delta was set to 1.15. This produced an FDR of 3.58%, and resulted in a list of 215 genes, of which an estimated 20 genes are falsely declared to be differentiated. A SAM plot is given in Figure 4, with some of the genes labeled. A complete list is given in Supplementary Table S1.

**Figure 5:**
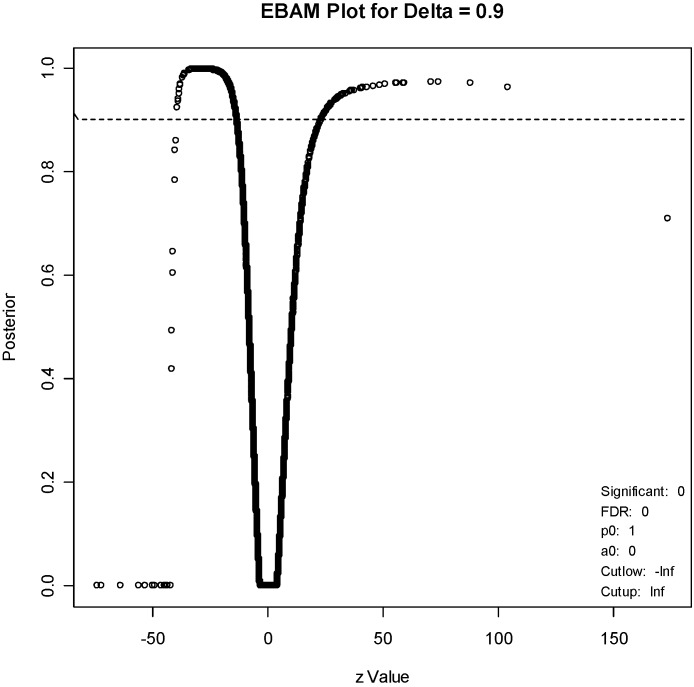
EBAM plot. The points above the dotted line correspond to genes with a posterior probability of 0.9 of being differentially expressed.

For the EBAM method, the required posterior probability was set to 0.7, which resulted in an FDR of 15.4%, to generate a list of about the same number of genes (211). An EBAM plot of posterior probabilities is given in Figure 5, and a complete list of differentially expressed genes is given in Supplementary Table S2. A list of the 61 genes that are present on both lists is given in Supplementary Table S3. One gene, with code “36711_at,” is clearly at the top of both lists. This protein is a novel MAFF (v-maf musculoaponeurotic fibrosarcoma (avian) oncogene family, protein F)-LIKE protein.

The diterpene connectivity scores for the 453 “instances” (representing treatment of various cell lines with 164 different compounds) are shown in Supplementary Tables S4 and S5. Common drug targets among the mostly highly connected instances include the proteasome, calmodulin, and histone deacetylases (HDACs). The presence of structurally distinct compounds that yield high connectivity scores suggests that the diterpene **1** acts as a proteasome and calmodulin inhibitor. Conversely, instances associated with the most highly ranked negative scores (corresponding to a gene expression profile similar to a reversal of the diterpene signature) include compounds that target the PI3 kinase/mTOR pathway. Additional inferences can be made from “permuted results” provided by the connectivity analysis (Table 1). In particular, analysis of permuted results supports the idea that the diterpene **1** is affecting cells in a manner similar to that of HSP90 and HDAC inhibitors and in a manner opposite to that of PI3 kinase/mTOR inhibitors. Note that the proteasome inhibitors are not included in the permuted results because they are represented by single instances.

**Table 1:** Permuted results.

Rank	CMAP name	Score	N	Enrichment	*P* value	Target
1	sirolimus	−0.254	10	−0.770	0.0000	mTOR
2	17-allylamino-geldanamycin	0.496	18	0.611	0.0000	HSP90
3	trichostatin A	0.565	12	0.714	0.0000	HDACs
4	LY-294002	−0.038	17	−0.506	0.0001	PI3 kinase
5	geldanamycin	0.544	6	0.759	0.0004	HSP90
6	5253409	0.748	2	0.971	0.0009	?
7	5224221	0.758	2	0.967	0.0015	?
8	vorinostat	0.684	2	0.956	0.0032	HDAC
9	tetraethylene-pentamine	−0.084	6	−0.662	0.0055	proteases
10	wortmannin	−0.248	8	−0.565	0.0071	PI3 kinase

Each row in the table summarizes the performance of all instances produced with the same perturbagen. The table displays the cmap name for those perturbagens, the arithmetic mean of the connectivity scores for each of those instances (labeled “score”), the number of those instances (labeled “*n*”), a measure of the enrichment of those instances in the list of all instances ordered by descending order of connectivity score and up score (labeled “enrichment”) and a permutation *p*-value for that enrichment score (labeled “*p*”). Enrichment scores and *p*-values are not provided for perturbagens represented by only one instance or where the mean of the connectivity scores for their instances is zero.

**Figure 6:**
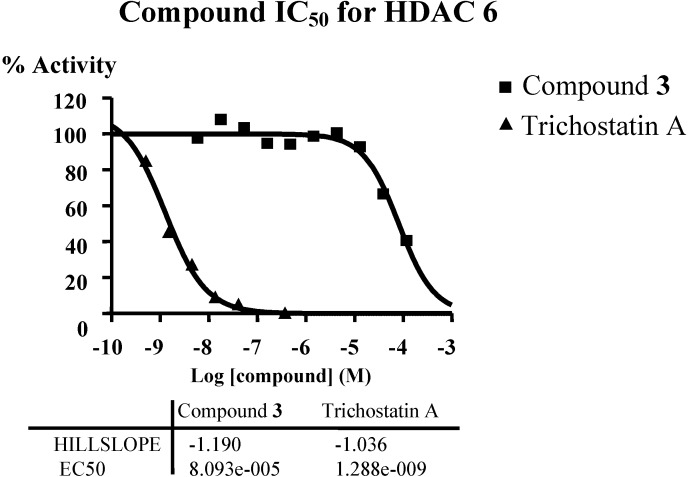
Inhibition of HDAC6 by compound **3**, IC_50_ around 80 µM.

In order to verify the predicted mode of action of diterpene from connectivity analysis, HDAC inhibitory assays were performed. Given the availability of compound material and the structural similarity of all isolated diterpenes, only compound **3** was subjected to enzymatic inhibition test against the class I (HDAC1, HDAC2, HDAC3 and HDAC8), the class II A (HDAC4, HDAC5, HDAC7 and HDAC9) and the class II B (HDAC6) enzymes. HDAC inhibitors Trichostatin A (TSA) and TMP269 (TMP) were used as reference compounds. The results showed that compound **3** up to 50 or 100 µM did not significantly inhibit the class I (HDAC1, Supplementary Table S6; HDAC2, Supplementary Table S7; HDAC3, Supplementary Table S8; HDAC8, Supplementary Table S9) as well as the class II A (HDAC4, Supplementary Table S10; HDAC5, Supplementary Table S11; HDAC7, Supplementary Table S12; HDAC9, Supplementary Table S13), though compound **3** inhibited class II B HDAC6 with IC_50_ around 80 µM (Figure 6, Supplementary Table S14). The predicted mode of action of diterpene was verified. HDAC6 appears to be the key deacetylase regulating Hsp90; treatment of breast cancer cells with Hsp90 inhibitors or HDAC inhibitor (vorinostat) induces breast cancer cell differentiation [17]. Cell death signaling is complex and it is clear that there are multiple alternate pathways, especially in cancer cell types. The HDAC6 inhibition pathway is among several pathways that the diterpene may trigger simultaneously, thus explaining the difference of potency between the apoptosis induction assay and the HDAC6 inhibition assay.

## 3. Experimental Section

### 3.1. General Experimental Procedures

Optical rotations were measured on JASCO P 1010 polarimeter. FT-IR spectra were obtained employing Hewlett Packard 8452A and Nicolet 510 instruments. All NMR spectra were recorded on a Bruker Avance DRX300 and DPX400 spectrometers. Spectra were referenced to residual solvent signal with resonances at δ_H/C_ 7.26/77.1 (CDCl_3_). ESI MS data were acquired on a Waters Micromass LCT Classic mass spectrometer and Varian 500-MS LC Ion Trap. HPLC separations were performed using Waters 510 HPLC pumps, a Waters 717 plus autosampler, and Waters 996 photodiode array detector. All solvents were purchased as HPLC grade.

### 3.2. Extraction and Isolation Procedures

The organism was purchased from Ocean Reef Aquariums (ORA). Live colonies of *Xenia elongata* were kept in optimal growing conditions (Salinity 34 g/L, temperature 25 °C, light [min. 250 µE m^−2^ s^−1^ and max. 500 µE m^−2^ s^−1^], pH 8.2–8.4) in the coral laboratory at the Marine Biotechnology Center, Institute of Marine and Coastal Sciences, Rutgers University. A voucher specimen is available as collection number SC/XE/Apr-04 System 1A. The soft coral was stored at −80 °C after addition of liquid nitrogen before workup. The material (80 g) was extracted three times, first with CH_2_Cl_2_ and then with MeOH, to give a nonpolar crude organic extract (259.2 mg) and a polar crude organic extract (537.9 mg). A portion of these two extracts (30 mg each) was tested for apoptosis induction. The polar crude organic extract was found active and subjected to fractionation by solid phase extraction cartridge (Reverse-phase C18) to give three fractions using a stepwise gradient of H_2_O-MeOH as solvent system. The fraction eluting with 15% H_2_O and 85% MeOH had apoptosis induction activity. This fraction was further chromatographed on analytical RP HPLC (Phenomenex luna C8, 250 × 4.60 mm) using a gradient elution (starting with 80% water and 20% CH_3_CN, flow rate 1 mL/mn) to yield successively 3.1 mg of **2** (*t*_R_ = 27 mn), 2.6 mg of **3** (*t*_R_ = 33 min). The extract/compound weight percents are 0.57% and 0.48% for **2** and **3**, respectively.

Compound **2**: [α]22.5 D −58 (*c* 0.87, CHCl_3_); IR ν_max_ (neat) 3075, 2970, 2930, 2860, 1736, 1670, 1608, 1445, 1370, 1236, 1155, 1015, 950, 870, 835, 790 cm^−1^; ^1^H NMR and ^13^C NMR, see Table 2; HRESIMS *m*/*z* 413.2299 (calcd. for C_23_H_34_O_5_Na, 413.2298).

**Table 2:** NMR Spectroscopic Data of Compound **2** ^1^H (300 MHz, CDCl_3_) and ^13^C (75 MHz, CDCl_3_).

Compound 2			
Position	δ_C_	δ_H_	HMBC
1	99.3 CH	5.35, d (1.4)	3, 4a
3	142.7 CH	6.51, s	1, 4, 4a, 12
4	112.6 qC		
4a	35.7 CH	2.19, dd (8, 12)	3, 4, 5, 11
5	31.0 CH_2_	1.50, m 1.95, m	4a, 6
6	40.1 CH_2_	2.08, m 2.22, m	5, 7, 8
7	135.5 qC		
8	124.6 CH	5.25, t (5.8)	9
9	24.7 CH_2_	2.45, m 2.08, m	8, 10
10	35.7 CH_2_	2.30, m	11, 19
11	150.0 qC		
11a	55.1 CH	1.70, dd (1.2,12)	1, 4a, 11
12	70.8 CH	4.85,dd (5.8, 6.1)	3, 4, 4a, 13, 14, CO
13	31.9 CH_2_	1.98, m 2.00, m	12, 14
14	62.8 CH	2.75, dd (5.9, 6.2)	13
15	60.1 qC		
16	18.9 CH_3_	1.33, s	14, 15, 17
17	24.7 CH_3_	1.33, s	14, 15, 16
18	17.7 CH_3_	1.60, s	6, 7, 8
19	119.8 CH_2_	5.05, s 5.15, s	10, 11, 11a
CH_3_ of CH_3_COO	21.0 CH_3_	2.09, s	CO
CO of CH_3_COO	170.9 qC		
CH_3_ of CH_3_O	51.5 CH_3_	3.52, s	1

Compound **3**: [α]^22.5^_D_ +12 (*c* 0.13, CHCl_3_); IR ν_max_ (neat) 3075, 2970, 1740, 1730, 1600, 1370, 1240 cm^−1^; ^1^H NMR and ^13^C NMR, see Table 3; HRESIMS *m*/*z* 365.2325 (calcd. for C_21_H_33_O_5_, 365.2328).

**Table 3:** NMR Spectroscopic Data of Compound **3** ^1^H (300 MHz, CDCl_3_) and ^13^C (75 MHz, CDCl_3_).

Compound 3			
Position	δ_C_	δ_H_	COSY
1	172.4 qC		
3	65.5 CH_2_	3.72, d(6)	
4	138.1 qC		
4a	38.3 CH	3.29, m	5, 11a
5	28.7 CH_2_	1.55, m 1.73, m	4a, 6
6	38.3 CH_2_	1.17, m 1.99, m	5
7	59.7 qC		
8	63.8 CH	2.80, dd (11.5, 3.2)	9
9	27.2 CH_2_	2.13, m 1.43, m	8, 10
10	26.2 CH_2_	2.13, m 2.34, m	9
11..	143.0 qC		
11a	60.6 CH	3.45, d (12.0)	4a
12	126.6 CH	5.45, dd (10.1, 7.2)	13
13	28.1 CH_2_	2.30, m 2.38, m	12, 14
14	63.3 CH	2.76, dd (7.2, 6.1)	13
15	58.8 qC		
16	19.0 CH_3_	1.29, s	
17	24.6 CH_3_	1.32, s	
18	18.8 CH_3_	1.21, s	
19	121.1 CH_2_	5.00, s 5.20, s	
CH_3_O	51.6 CH_3_	3.50, s	

### 3.3. Biological Evaluation–Apoptosis Induction

Apoptosis induction in the presence of compounds (**2**, **3**) was carried out in the following manner. W2 (apoptosis competent) and D3 (apoptosis defective) cells were plated in 96- and 6-well plates and incubated for 24 h, after which they were evenly spread at about 50% confluency. At this time, compounds dissolved in DMSO and diluted in growth medium (DMEM) were added to the cells at various concentrations. DMSO concentration was kept at 0.5% in all wells. Plates were incubated for 24, 48 and 72 h. Cell viability was determined using a modification of the MTT assay [11], where the reduction of yellow tertazolium salt (MTT—3-(4,5-dimethylthiazol-2-yl)-2,5) to purple formazan indicates mitochondrial activity, and thus cell viability. Cells were incubated with 0.5 mg/mL MTT for 3 h. Supernatant was aspirated and DMSO was added to dissolve the formazan crystals. After 30 min incubation at 37 °C, with shaking, absorbance was read at 570 nm on a Spectra MAX 250 (Molecular Devices) plate reader. Differential growth from time 0 to 48 h was calculated. Starurosporine, an apoptosis inducer, and DMSO were used as positive and negative controls, respectively.

### 3.4. Connectivity Analyses

Cells were harvested and RNA extracted using TRIzol Reagent, (Invitrogen, Carlsbad, CA, USA) and RNeasy kit (Qiagen Sciences, MD, USA). RNA was purified, reverse transcribed, and hybridized to an Affymetrix U133 2.0 array by the CINJ Transcriptional Profiling Core. The resulting gene expression data were analyzed. Two methods were used to identify the most differentially expressed genes in the treated *versus* control groups. The first method (significance analysis of arrays) was set at a false discovery rate of 3.58%, and yielded 215 genes. A subset of these 215 genes, including 145 with at least a 3-fold difference in expression (58 upregulated by treatment, 87 downregulated), was defined as the diterpene signature, and was used for the connectivity analysis supplementary (Supplementary Table S5). The diterpene signature was compared to 453 gene expression profiles present in the Connectivity Map [13]. For each of the 453 profiles, a connectivity score was calculated (based on the Kolmogorov–Smirnov statistic), which represents the relative similarity of the profile to the diterpene signature.

### 3.5. HDAC Inhibition Assay

#### 3.5.1. Class I

In singlet 10-dose IC_50_ mode with 3-fold serial dilution starting at 50 µM against 4 HDACs (HDAC1, HDAC2, HDAC3 and HDAC8), 0.5 mg of Compound **3** was tested. 

HDAC reference compound Trichostatin A (TSA) and TMP 269 were tested in a 10-dose IC_50_ with 3-fold serial dilution starting at 10 μM.

Substrate for HDAC1, 2 and 3: Fluorogenic peptide from p53 residues 379–382 (RHKK(Ac)AMC.

Substrate for HDAC8: Fluorogenic peptide from p53 residues 379–382 (RHK(Ac)K(Ac)AMC).

Data pages include raw data, % Enzyme activity (relative to DMSO controls), and curve fits. Curve fits were performed where the enzyme activities at the highest concentration of compounds were less than 65%. IC_50_ values were calculated using the GraphPad Prism 4 program based on a sigmoidal dose-response equation. The blank (DMSO) value was entered as 1.0 × 10^−12^ of concentration for curve fitting.

#### 3.5.2. Class II A and Class II B

In singlet 10-dose IC_50_ mode with 3-fold serial dilution starting at 100 µM against 5 HDACs [II A (HDAC4, HDAC5, HDAC7, and HDAC9) IIB (HDAC6)], 0.5 mg of Compound **3** was tested. 

HDAC reference compounds Trichostatin A (TSA) and TMP269 (TMP) were tested in a 10-dose IC_50_ with 3-fold serial dilution starting at 10 µM.

Substrate for HDAC 6: Fluorogenic peptide from p53 residues 379–382 (RHKK(Ac)AMC.

Substrate for HDAC 4,5,7 and 9: Fluorogenic HDAC Class2a Substrate (Trifluoroacetyl Lysine, Ac-LGK(TFA)-AMC).

IC_50_ values were calculated using the GraphPad Prism 4 program based on a sigmoidal dose-response equation. The blank (DMSO) value was entered as 1.0 × 10^−12^ of concentration for curve fitting. 

#### 3.5.3. Class II B for IC_50_ Calculation

0.5 mg of Compound **3** was tested in duplicate 10-dose IC_50_ mode with 3-fold serial dilution starting at 120 µM against 1 HDAC (HDAC6).

HDAC reference compounds Trichostatin A (TSA) was tested in a 10-dose IC_50_ with 3-fold serial dilution starting at 10 µM.

Substrate for HDAC6: Fluorogenic peptide from p53 residues 379–382 (RHKK(Ac)AMC.

IC_50_ values were calculated using the GraphPad Prism 4 program based on a sigmoidal dose-response equation. The blank (DMSO) value was entered as 1.0 × 10^−12^ of concentration for curve fitting.

## 4. Conclusions

The compounds described here represent novel natural products with specific proapoptotic, and therefore potential anticancer, activities. This work describes the mode of action of diterpene from the soft coral *Xenia elongata* using connectivity analysis for MCF-7 cells treated with a coral diterpene. The coral diterpene is affecting cells in a manner similar to that of HSP90 and HDAC inhibitors and in a manner opposite to that of PI3 kinase/mTOR inhibitors. The vast majority of human solid tumors are of epithelial origin, and defects in apoptosis, mostly upstream of Bax and Bak, play important roles in both tumor suppression and mediation of chemotherapeutic response. Consequently, efforts are increasingly focused on developing drugs that can re-activate the apoptotic pathway. The compounds identified here induce apoptosis upstream of Bax and Bak and may have a potential for use as anticancer agents that exploit the apoptosis pathway in tumor cells. Furthermore, the diterpene selectively inhibits HDAC6 and represents a new model structure of selective HDAC inhibitors which will contribute to the development of HDAC practical isoform selective [18]. Future work will likely focus on the identification of the protein targeted by the coral diterpene and the optimization of essential pharmocophores.

## Supplementary Files

Supplementary File 1 Supplementary Information (PDF, 2038 KB)
