# Supplementary material for: Mode of Action of Diterpene and Characterization of Related Metabolites from the Soft Coral, Xenia elongata
Source: Mar Drugs. 2014 Feb 20;12(2):1102–15. doi: 10.3390/md12021102 (PMC3944532; doi:10.3390/md12021102)
Supplement: Supplementary File 1 — Supplementary Information (PDF, 2038 KB) [file marinedrugs-12-01102-s001.pdf]

Supplementary Information

Figure S1. 300 MHz ^{1}H NMR spectrum of compound (**2**) in $CDCl_3$.

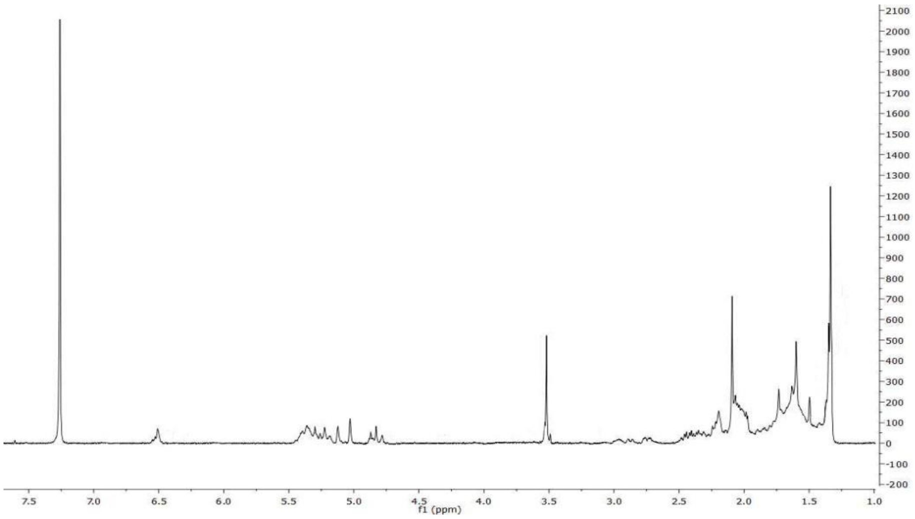

Figure S2.75 MHz ^{13}C NMR spectrum of compound (**2**) in $CDCl_3$.

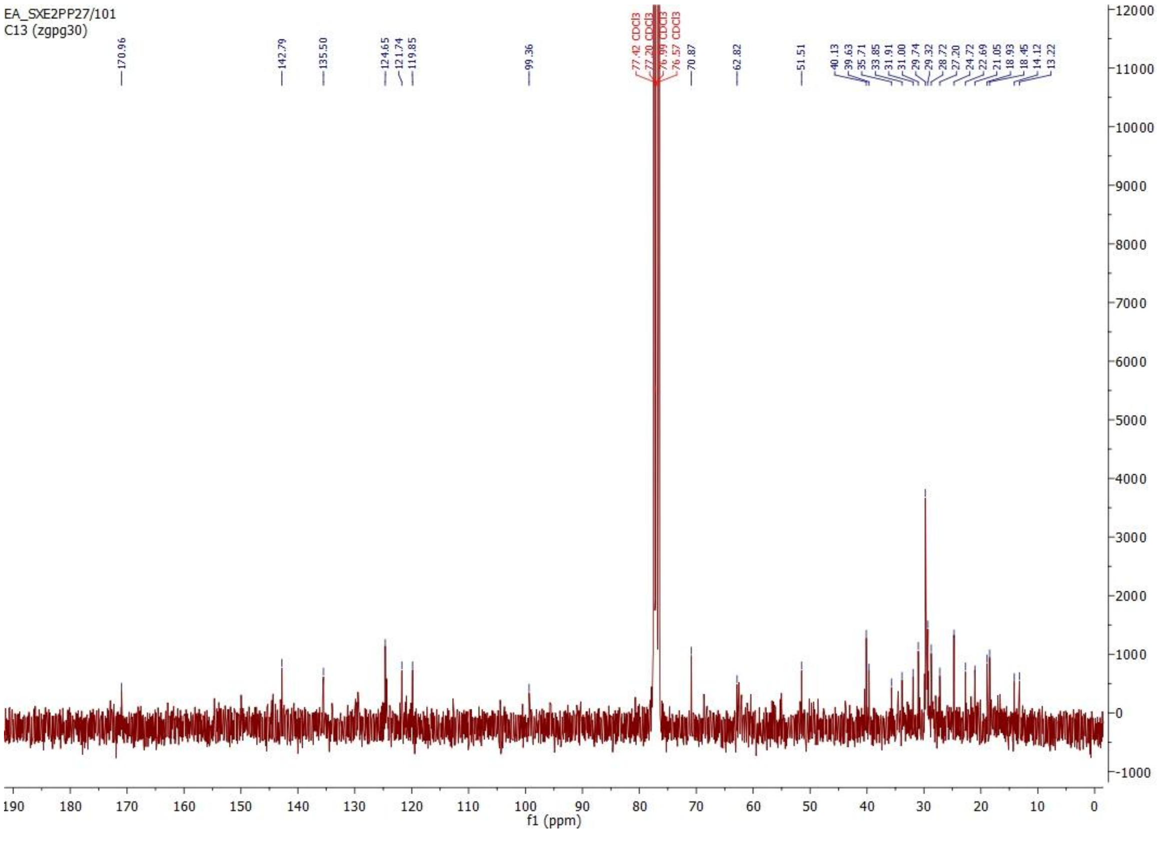

Figure S3. 300 MHz ROESY NMR spectrum of compound (**2**) in $CDCl_3$.

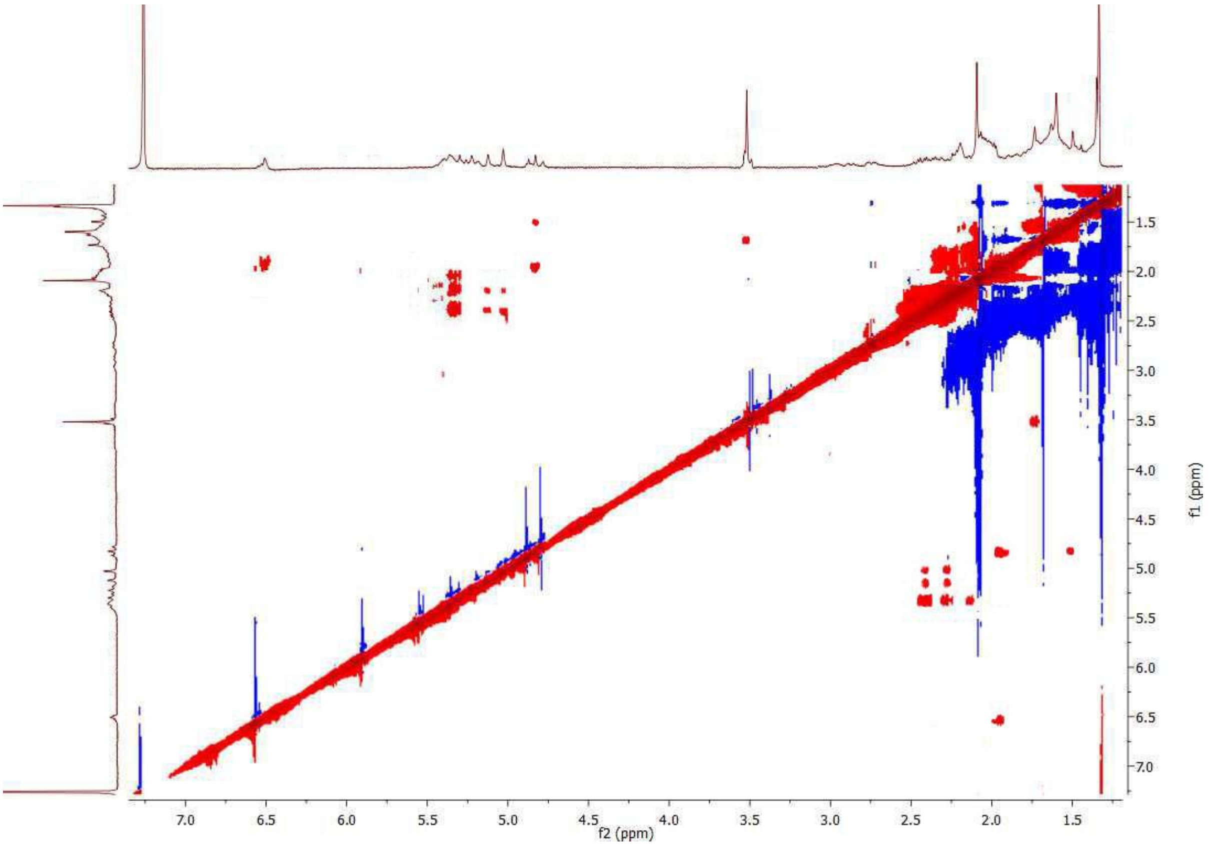

Figure S4. Low Resolution ESI-MS spectrum of compound (**3**).

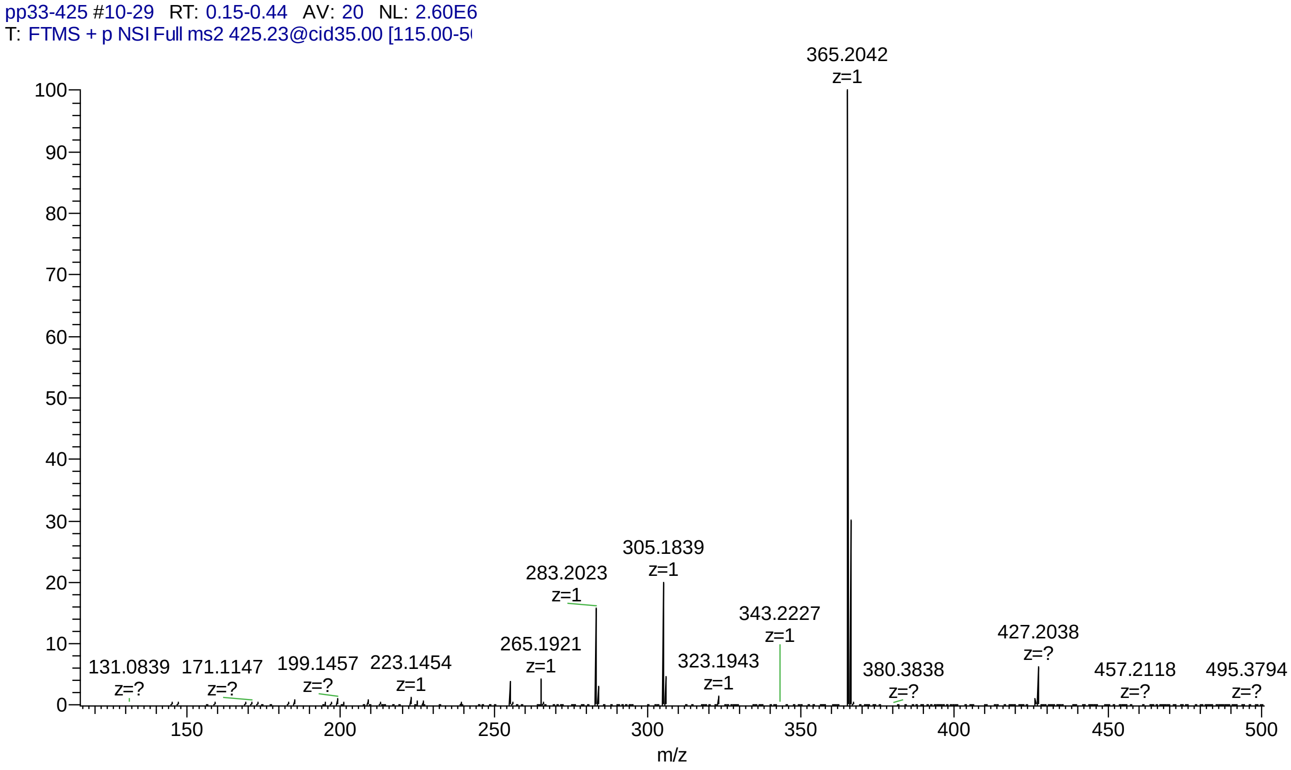

Figure S5. 300 MHz ^{1}H NMR spectrum of compound (**3**) in $CDCl_3$.

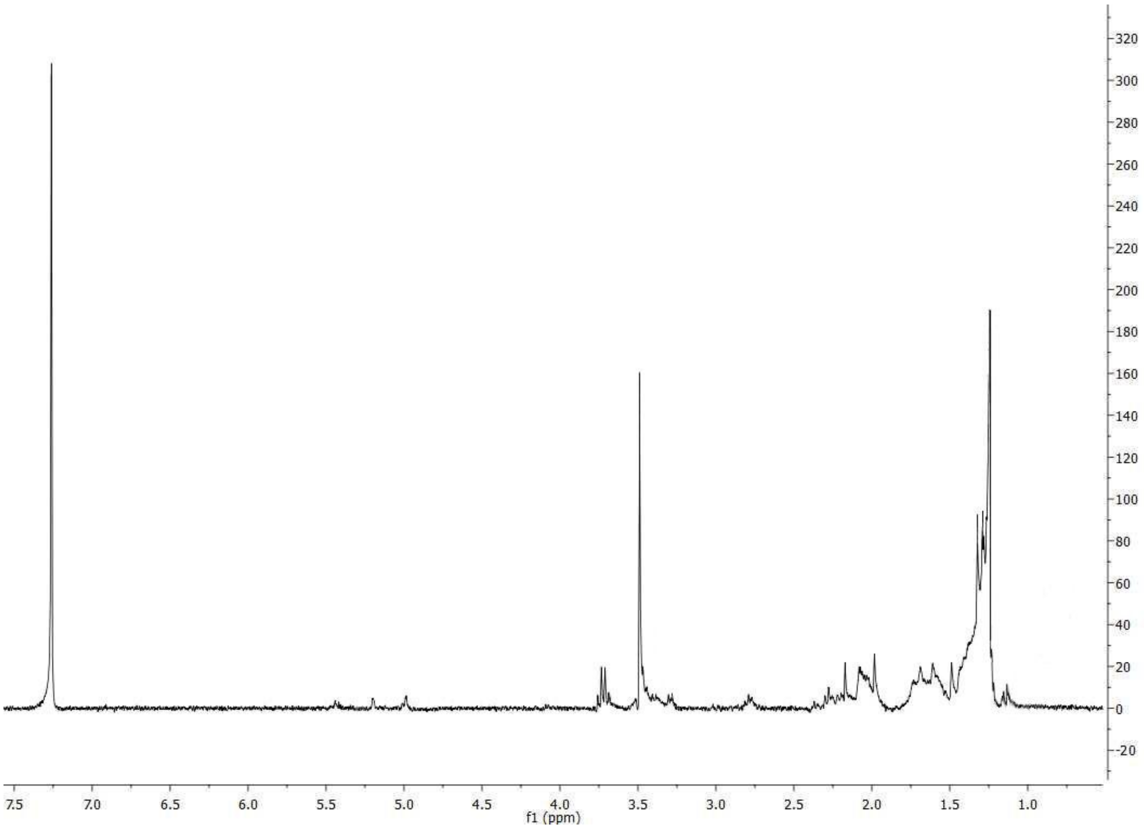

Figure S6. 75 MHz ^{13}C NMR spectrum of compound (**3**) in $CDCl_3$.

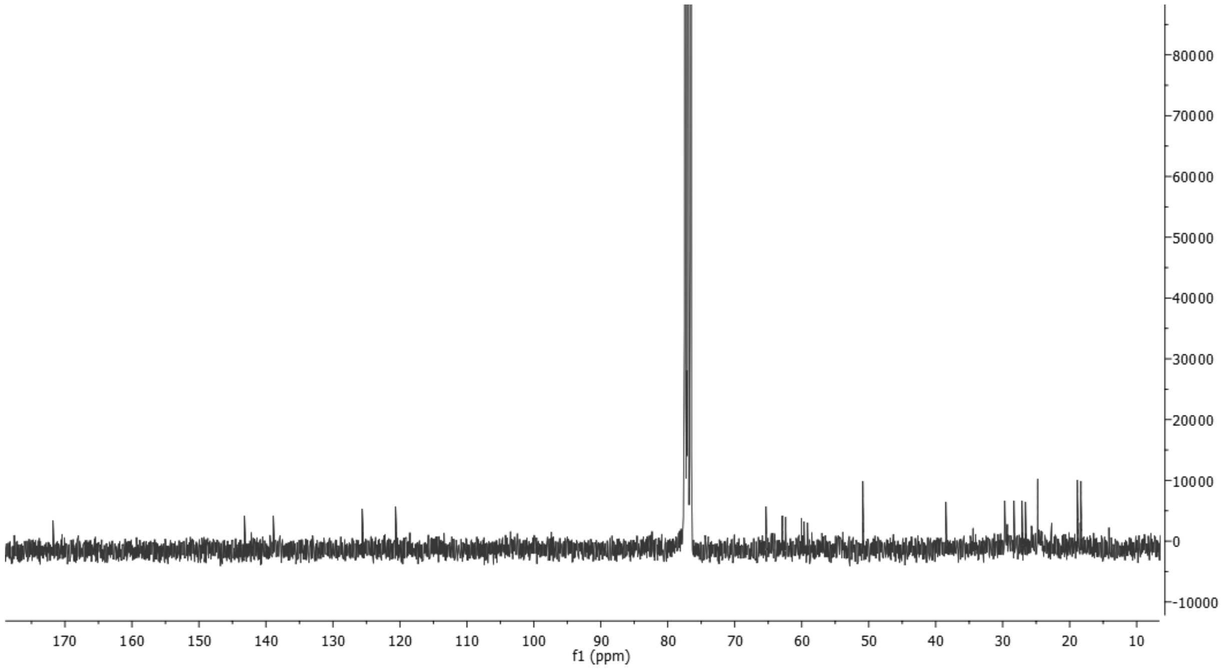

Figure S7. 300 MHz ROESY (partial view) NMR spectrum of compound (**3**) in $CDCl_3$.

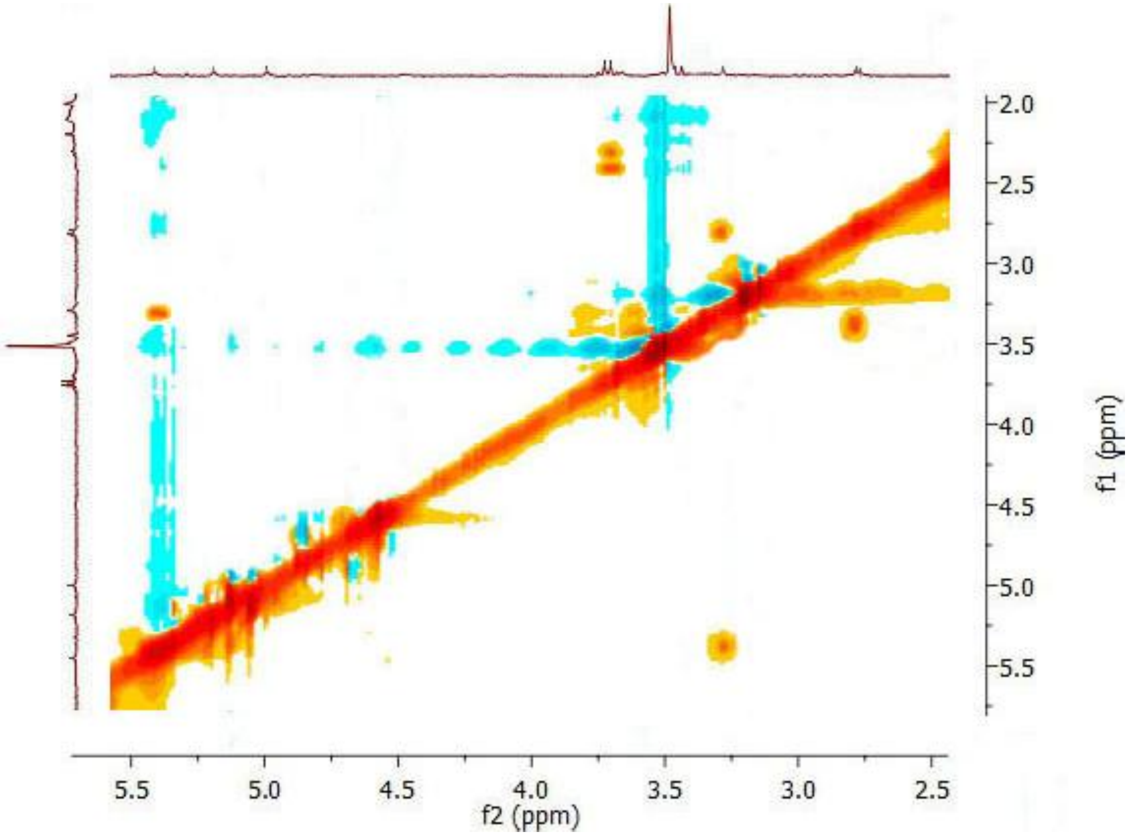

Figure S8. 300 MHz COSY NMR spectrum of compound (**2**) in $CDCl_3$.

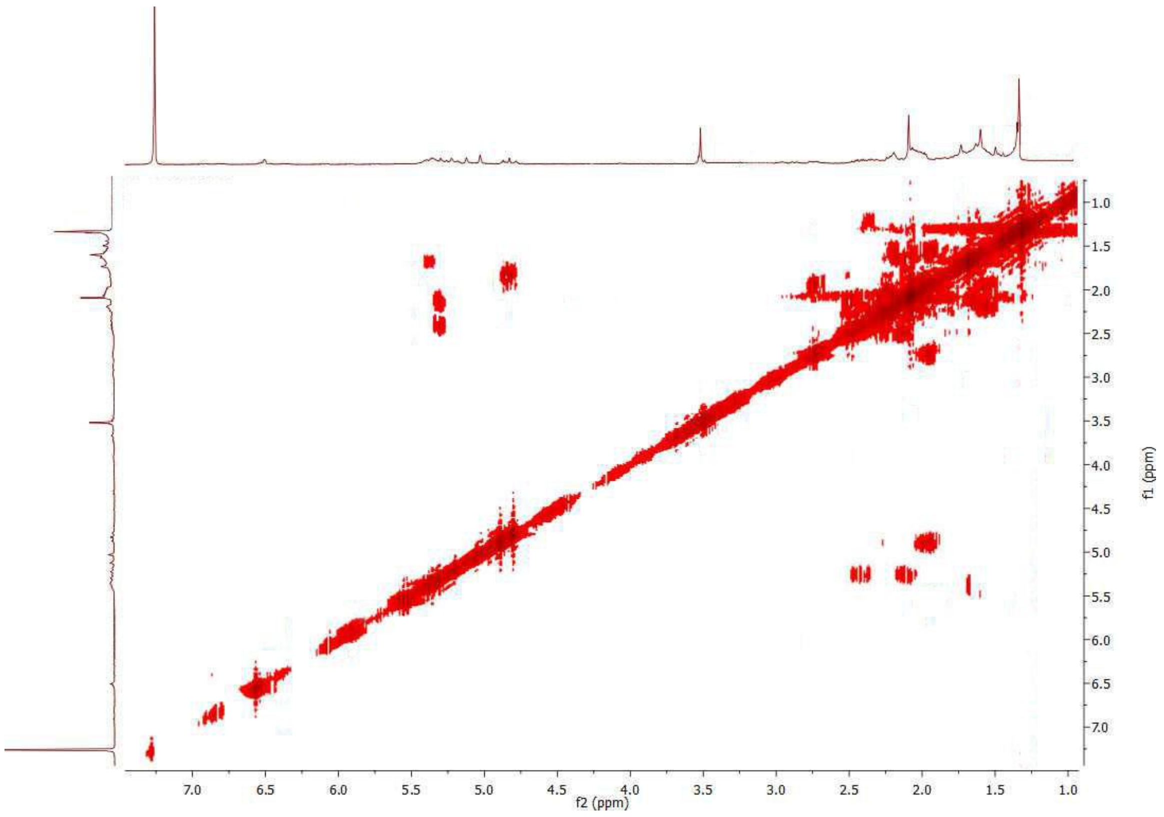

Figure S9. 300 MHz Multiplicity edited HSQC NMR spectrum of compound (**2**) in $CDCl_3$.

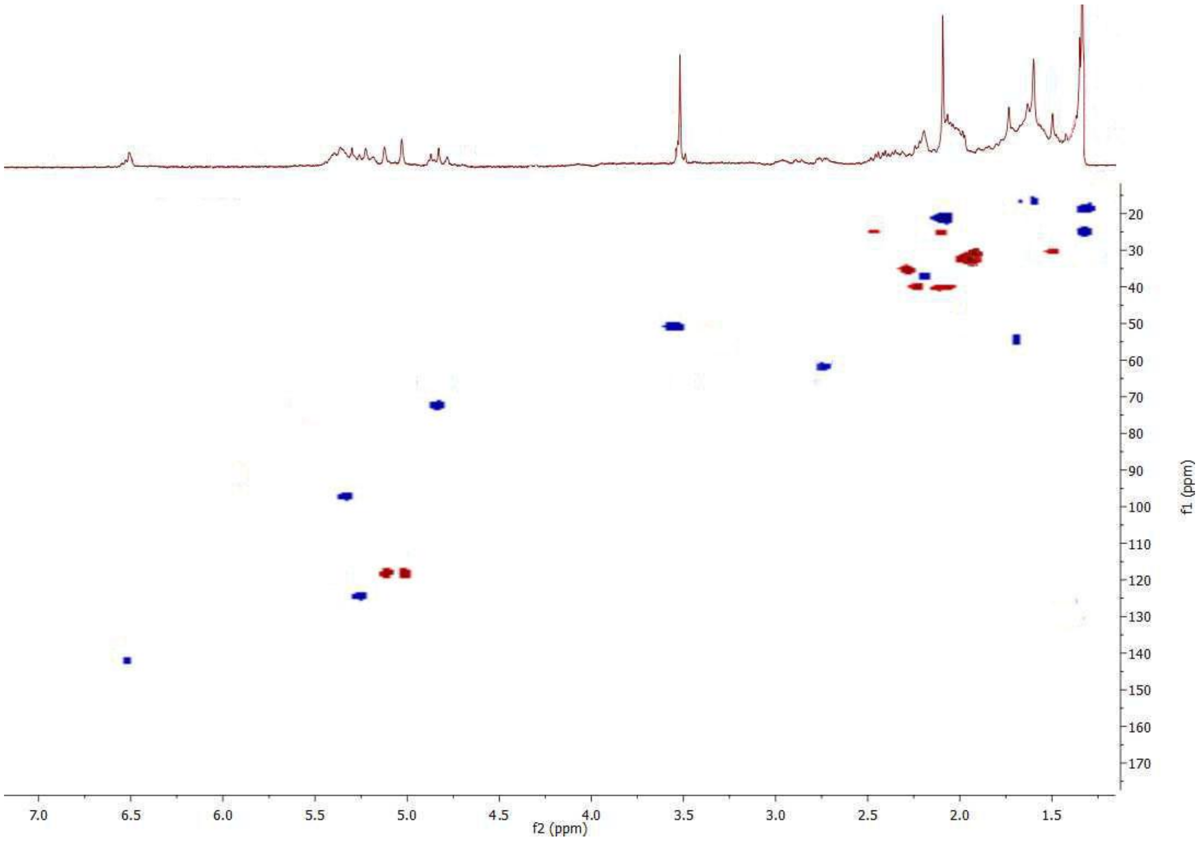

Figure S10. 300 MHz HMBC NMR spectrum of compound (**2**) in $CDCl_3$.

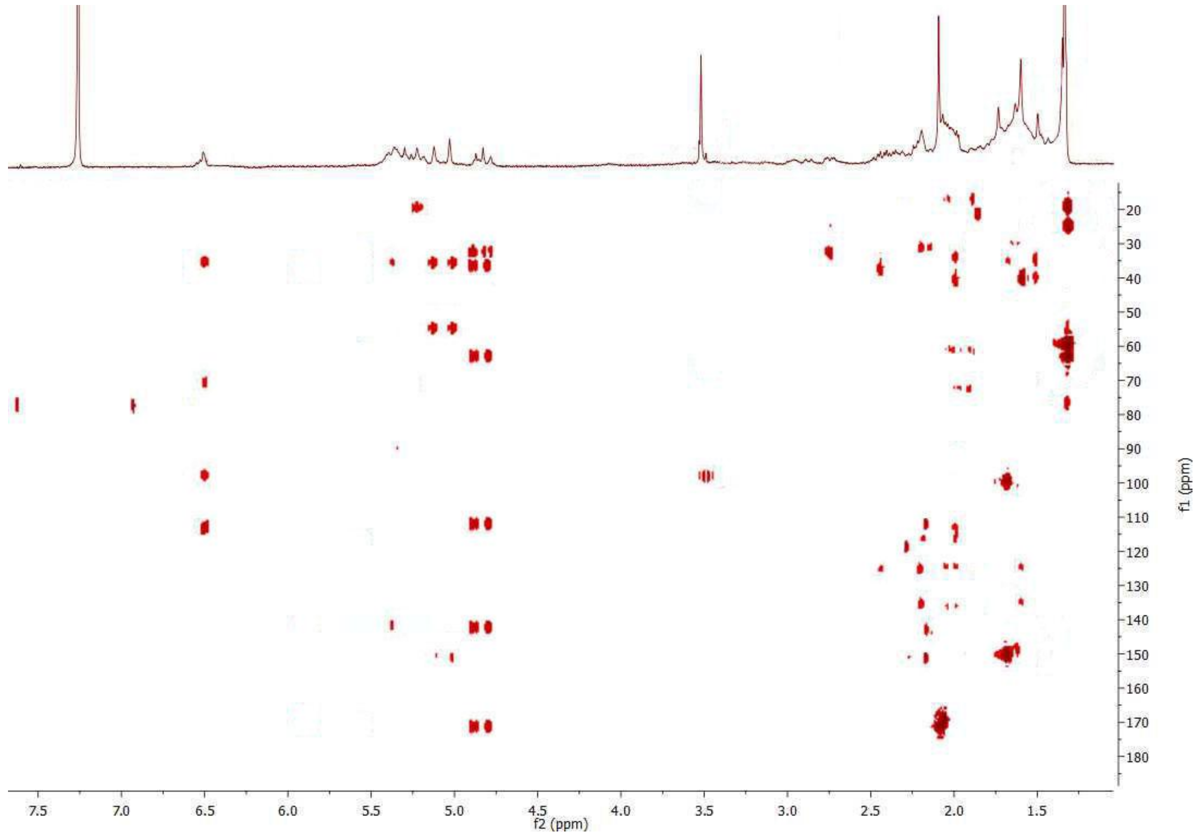

Figure S11. 300 MHz COSY spectrum of compound (**3**) in $CDCl_3$.

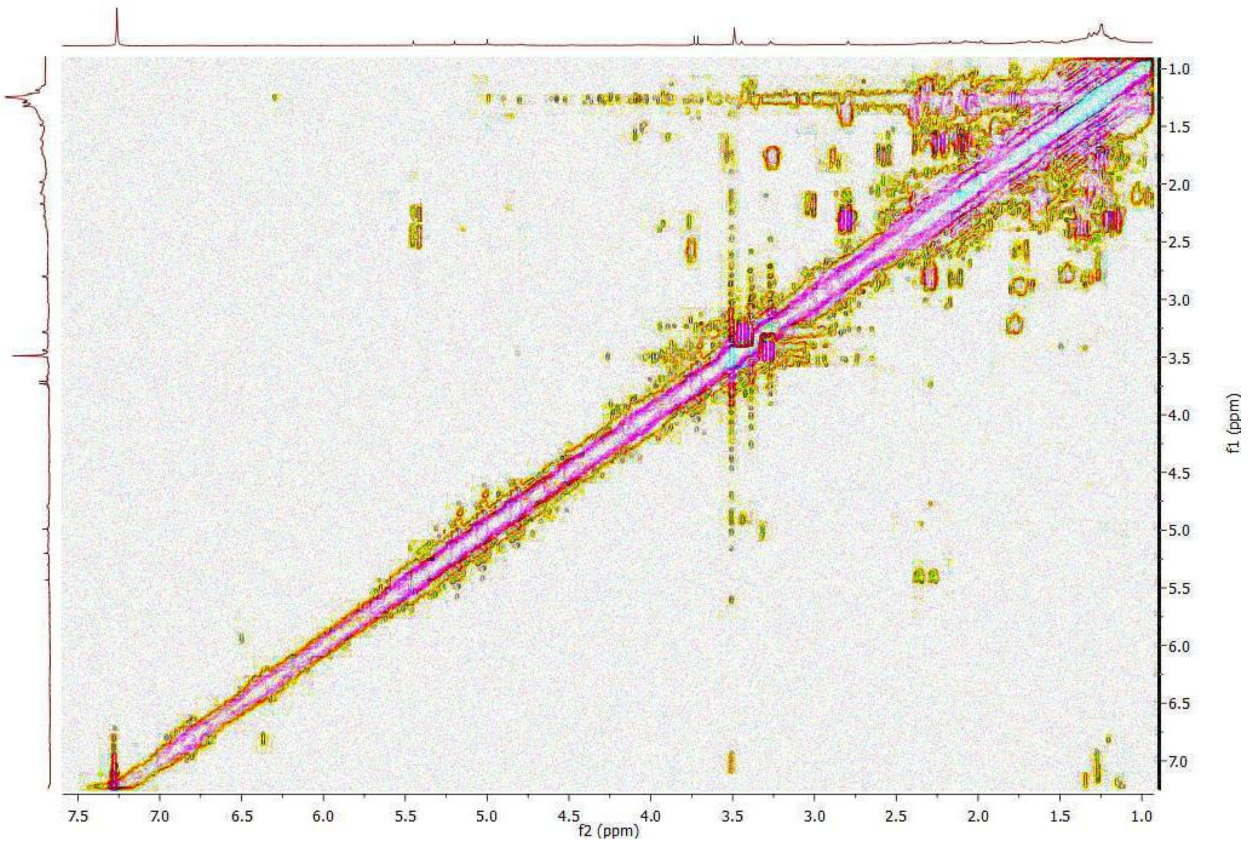

Figure S12. 300 MHz Multiplicity edited HSQC NMR spectrum of compound (**3**) in $CDCl_3$.

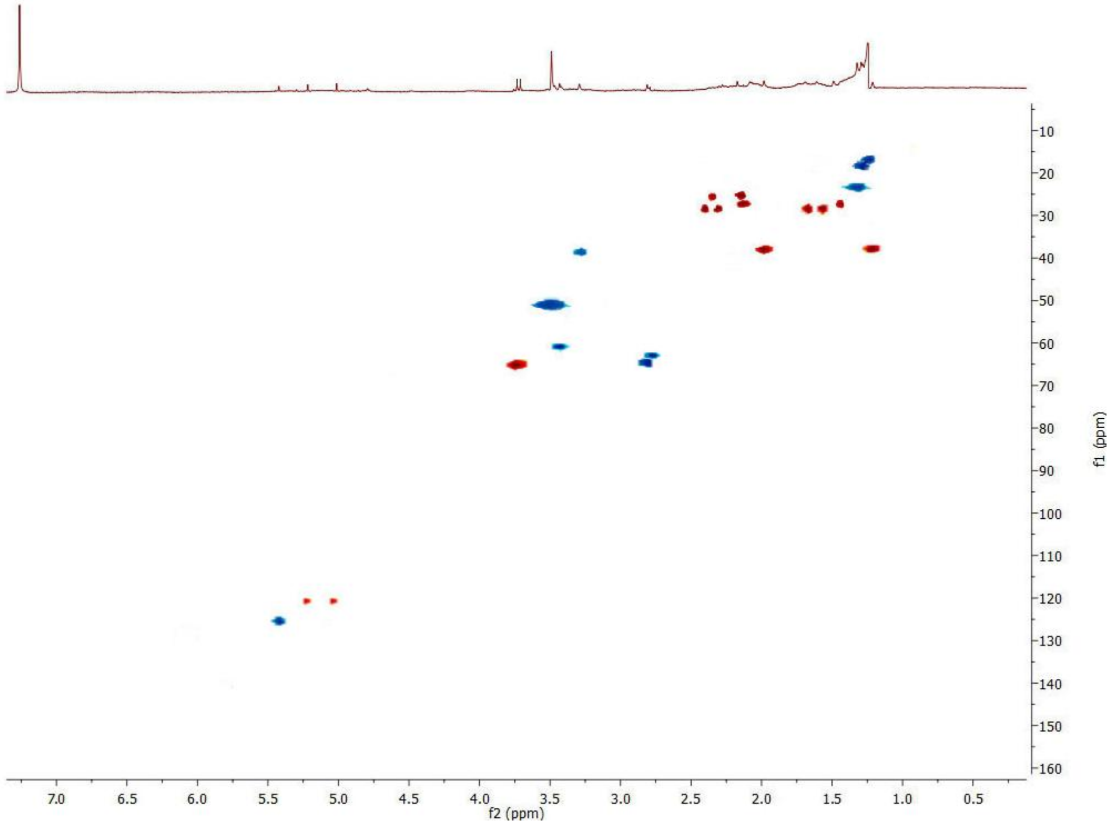

Figure S13. 300 MHz HMBC NMR spectrum of compound (**3**) in $CDCl_3$.

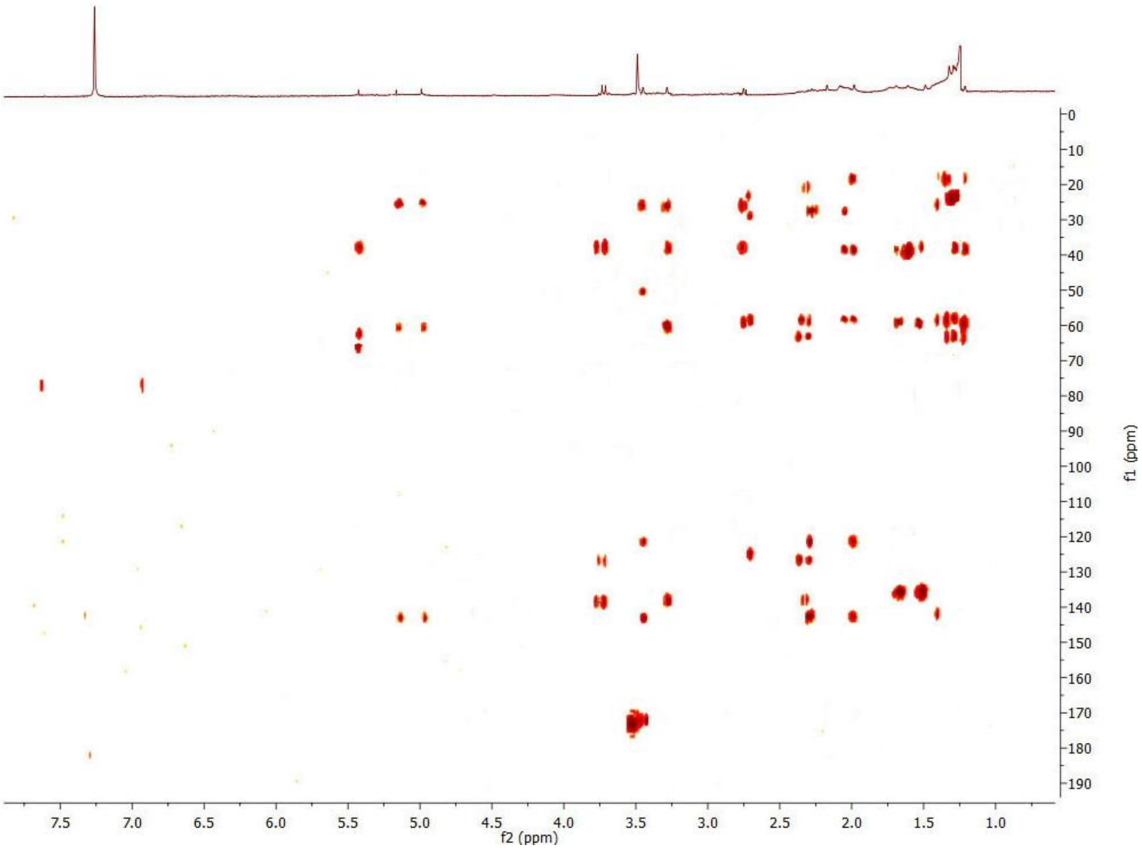

Figure S14. Biological activity apoptosis induction assay for compound (**2**).

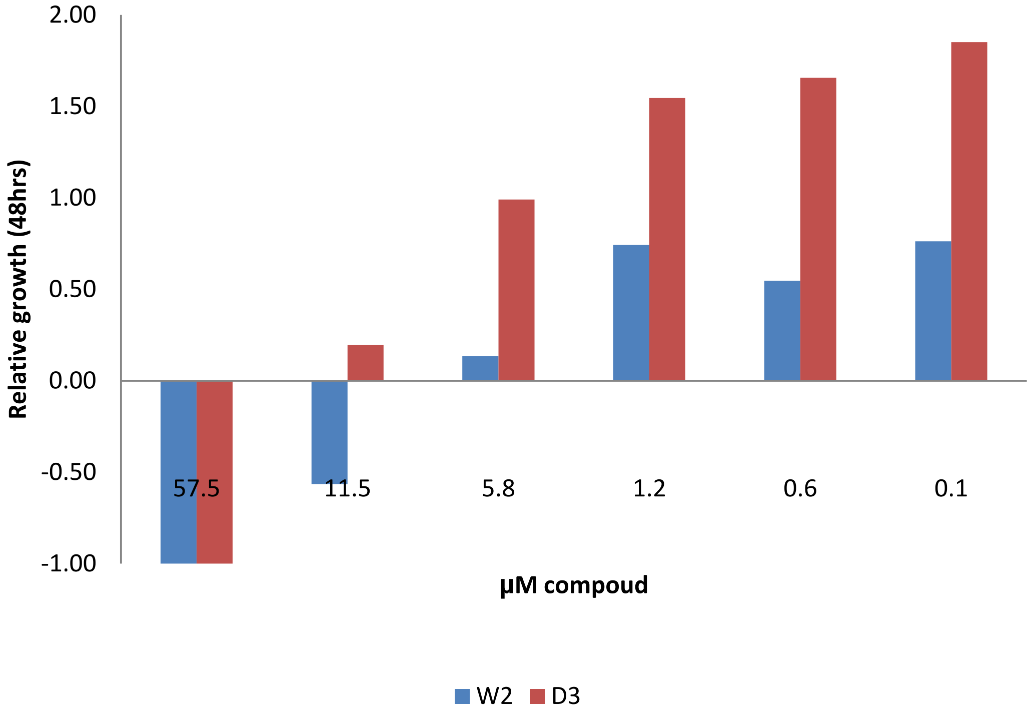

Figure S15. Biological activity apoptosis induction assay for compound (**3**).

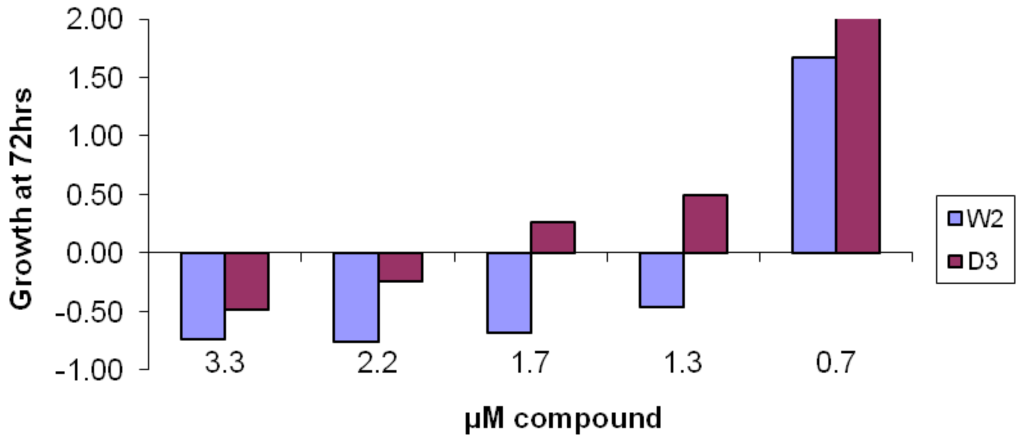

Table S1. Differentially-expressed genes using the SAM method. The raw (unadjusted) *p*-value and the fold-difference is given for each gene. Also given is the "d.value" (the modified *t*-test statistic) and its standard error.

Identified Genes (using Delta = 1.15):

	Row	d.value	stdev	rawp	q.value	R.fold	Name
1	11114	6.87	0.0288	8.77E-06	0.0381	32.063	36711_at
2	1966	6.5	0.1776	1.75E-05	0.0381	51.404	202672_s_at
3	2718	6.2	0.1182	2.63E-05	0.0381	33.809	203665_at
4	5123	5.94	0.1183	3.51E-05	0.0381	28.912	209383_at
5	1421	5.67	0.1569	4.39E-05	0.0381	29.246	202014_at
6	11125	5.34	0.0453	5.26E-05	0.0381	15.798	37028_at
7	3208	4.79	0.0485	6.14E-05	0.0381	12.023	204472_at
8	1888	4.37	0.0592	7.02E-05	0.0381	10.005	202581_at
9	2888	4.14	0.1084	7.89E-05	0.0381	10.127	203946_s_at
10	213	4.01	0.0766	8.77E-05	0.0381	8.685	200664_s_at
11	9446	3.86	0.0726	9.65E-05	0.0381	7.895	218723_s_at
12	9356	3.47	0.0242	0.000105	0.0381	5.721	218611_at
13	2817	3.37	0.2171	0.000114	0.0381	8.395	203810_at
14	937	3.26	0.1	0.000123	0.0381	6.078	201466_s_at
15	1146	3.18	0.073	0.000132	0.0381	5.473	201694_s_at
16	4983	3.16	0.1934	0.00014	0.0381	6.972	209189_at
17	4275	3.1	0.1178	0.000149	0.0381	5.779	207714_s_at
18	214	3.09	0.0994	0.000158	0.0381	5.563	200666_s_at
19	3341	-3.08	0.0532	0.000167	0.0381	0.2	204686_at
20	565	3.04	0.0969	0.000175	0.0381	5.347	201041_s_at
21	4600	3.02	0.038	0.000184	0.0381	4.698	208744_x_at
22	10380	-3	0.1291	0.000193	0.0381	0.178	220770_s_at
23	8676	-2.97	0.1117	0.000202	0.0381	0.188	217853_at
24	3303	2.96	0.1077	0.000211	0.0381	5.232	204621_s_at
25	936	2.93	0.0389	0.000219	0.0381	4.493	201464_x_at
26	7556	2.9	0.0662	0.000228	0.0381	4.677	213988_s_at
27	10406	-2.9	0.0994	0.000237	0.0381	0.201	220940_at

28	341	2.84	0.0464	0.000246	0.0381	4.341	200800_s_at
29	2759	2.82	0.0715	0.000254	0.0381	4.521	203725_at
30	7976	-2.79	0.0896	0.000263	0.0381	0.218	215073_s_at
31	6394	-2.78	0.1773	0.000272	0.0381	0.183	212239_at
32	6404	-2.74	0.0869	0.000281	0.0381	0.224	212249_at
33	3912	-2.7	0.045	0.000289	0.0381	0.248	206115_at
34	11309	-2.66	0.1035	0.000298	0.0381	0.227	55872_at
35	1807	-2.66	0.0777	0.000307	0.0381	0.239	202478_at
36	5684	2.66	0.087	0.000316	0.0381	4.266	210592_s_at
37	2563	2.65	0.1518	0.000325	0.0381	4.739	203455_s_at
38	3109	2.63	0.0768	0.000333	0.0381	4.119	204285_s_at
39	7808	2.61	0.0732	0.000342	0.0381	4.045	214683_s_at
40	1254	-2.59	0.2343	0.000351	0.0381	0.19	201818_at
41	4102	2.59	0.0651	0.00036	0.0381	3.942	206976_s_at
42	3110	2.57	0.1322	0.000368	0.0381	4.376	204286_s_at
43	2098	2.56	0.1385	0.000377	0.0381	4.406	202843_at
44	3291	-2.55	0.0565	0.000386	0.0381	0.263	204602_at
45	6973	-2.54	0.0751	0.000395	0.0381	0.256	212977_at
46	9959	2.53	0.1771	0.000404	0.0381	4.651	219475_at
47	10304	-2.52	0.2415	0.000412	0.0381	0.197	220391_at
48	10285	-2.51	0.0442	0.000421	0.0381	0.274	220305_at
49	4930	-2.5	0.0643	0.00043	0.0381	0.267	209120_at
50	4637	2.49	0.0459	0.000439	0.0381	3.623	208786_s_at
51	8415	-2.46	0.0692	0.000447	0.0381	0.269	217122_s_at
52	10922	-2.46	0.0392	0.000456	0.0381	0.283	222108_at
53	4259	2.44	0.0602	0.000465	0.0381	3.606	207630_s_at
54	11373	2.42	0.115	0.000474	0.0381	3.932	65588_at
55	1535	2.4	0.0505	0.000482	0.0381	3.481	202147_s_at
56	3727	-2.39	0.0973	0.000491	0.0381	0.266	205527_s_at
57	4842	2.39	0.0296	0.0005	0.0381	3.352	209020_at
58	7013	-2.39	0.0467	0.000509	0.0381	0.29	213032_at
59	8420	2.39	0.0843	0.000518	0.0381	3.667	217144_at
60	5271	-2.38	0.0933	0.000526	0.0381	0.27	209602_s_at
61	9873	-2.36	0.0806	0.000535	0.0381	0.279	219330_at
62	10757	-2.36	0.1419	0.000544	0.0381	0.251	221786_at
63	2522	-2.36	0.0308	0.000553	0.0381	0.302	203395_s_at
64	6781	2.36	0.0667	0.000561	0.0381	3.513	212722_s_at
65	4116	-2.35	0.0448	0.00057	0.0381	0.297	207038_at
66	9845	-2.35	0.0995	0.000579	0.0381	0.273	219283_at
67	8388	-2.34	0.1076	0.000588	0.0381	0.27	216971_s_at
68	7003	-2.34	0.0932	0.000596	0.0381	0.276	213019_at
69	6782	2.33	0.0878	0.000605	0.0381	3.564	212723_at
70	7247	-2.32	0.0752	0.000614	0.0381	0.288	213390_at
71	2855	-2.32	0.0998	0.000623	0.0381	0.277	203887_s_at
72	4110	-2.31	0.1432	0.000632	0.0381	0.261	207012_at
73	2948	-2.26	0.1029	0.00064	0.0381	0.284	204029_at
74	6986	-2.26	0.1488	0.000649	0.0381	0.266	212993_at

75	8833	-2.25	0.046	0.000658	0.0381	0.312	218019_s_at
76	3262	-2.25	0.0934	0.000667	0.0381	0.292	204554_at
77	7581	-2.24	0.2699	0.000675	0.0381	0.229	214046_at
78	4114	2.23	0.1061	0.000684	0.0381	3.486	207030_s_at
79	2149	2.22	0.1652	0.000693	0.0381	3.755	202912_at
80	8604	-2.2	0.0408	0.000702	0.0381	0.323	217779_s_at
81	6395	-2.2	0.1103	0.000711	0.0381	0.291	212240_s_at
82	1534	2.2	0.0792	0.000719	0.0381	3.294	202146_at
83	3304	2.2	0.0907	0.000728	0.0381	3.335	204622_x_at
84	2097	2.2	0.0445	0.000737	0.0381	3.115	202842_s_at
85	3039	-2.19	0.1385	0.000746	0.0381	0.278	204172_at
86	10688	-2.19	0.0359	0.000754	0.0381	0.327	221688_s_at
87	3734	2.18	0.1722	0.000763	0.0381	3.709	205543_at
88	855	-2.18	0.0636	0.000772	0.0381	0.316	201368_at
89	3961	-2.18	0.1101	0.000781	0.0381	0.295	206373_at
90	3147	-2.16	0.1034	0.000789	0.0381	0.3	204352_at
91	6297	-2.16	0.098	0.000798	0.0381	0.304	212126_at
92	4364	2.16	0.0532	0.000807	0.0381	3.082	208078_s_at
93	10625	2.16	0.079	0.000816	0.0381	3.212	221577_x_at
94	10154	-2.16	0.119	0.000825	0.0381	0.296	219918_s_at
95	9747	-2.15	0.0878	0.000833	0.0381	0.308	219127_at
96	8257	2.15	0.1319	0.000842	0.0381	3.461	216248_s_at
97	340	2.15	0.0504	0.000851	0.0381	3.058	200799_at
98	7930	-2.15	0.0979	0.00086	0.0381	0.306	214949_at
99	5249	-2.14	0.2355	0.000868	0.0381	0.25	209566_at
100	9516	2.14	0.1567	0.000877	0.0381	3.538	218810_at
101	7075	-2.14	0.1244	0.000886	0.0381	0.296	213117_at
102	10811	-2.13	0.163	0.000895	0.0381	0.282	221869_at
103	2690	-2.13	0.0514	0.000904	0.0381	0.33	203628_at
104	6447	-2.12	0.1025	0.000912	0.0381	0.307	212298_at
105	2733	-2.12	0.1305	0.000921	0.0381	0.296	105 203685_at
106	7078	-2.12	0.0596	0.00093	0.0381	0.328	213122_at
107	1359	-2.12	0.0206	0.000939	0.0381	0.347	201939_at
108	5334	-2.12	0.0375	0.000947	0.0381	0.339	209711_at
109	6337	2.11	0.0316	0.000956	0.0381	2.909	212171_x_at
110	3729	-2.1	0.1323	0.000965	0.0381	0.298	205534_at
111	5654	2.1	0.0768	0.000974	0.0381	3.097	210512_s_at
112	3927	-2.1	0.2497	0.000982	0.0381	0.254	206188_at
113	11010	-2.09	0.1493	0.000991	0.0381	0.294	222313_at
114	783	2.09	0.2171	0.001	0.0381	3.705	201289_at
115	2301	-2.09	0.0796	0.001009	0.0381	0.324	203123_s_at
116	7815	2.08	0.116	0.001018	0.0381	3.235	214696_at
117	10827	-2.07	0.1363	0.001026	0.0381	0.3	221892_at
118	6336	-2.07	0.1792	0.001035	0.0381	0.287	212170_at
119	9708	-2.07	0.1886	0.001044	0.0381	0.284	219069_at
120	899	-2.07	0.0403	0.001053	0.0381	0.346	201417_at
121	7148	-2.06	0.0817	0.001061	0.0381	0.327	213233_s_at

122	9275	-2.06	0.152	0.00107	0.0381	0.299	218519_at
123	4083	2.05	0.2565	0.001079	0.0381	3.809	206907_at
124	8426	2.04	0.0207	0.001088	0.0381	2.775	217168_s_at
125	1736	-2.04	0.1154	0.001096	0.0381	0.314	202392_s_at
126	11106	-2.04	0.0795	0.001105	0.0381	0.333	36499_at
127	459	2.03	0.0746	0.001114	0.0381	2.986	200924_s_at
128	7271	-2.03	0.0919	0.001123	0.0381	0.329	213435_at
129	10858	-2.01	0.0362	0.001132	0.0381	0.358	221963_x_at
130	2439	-2.01	0.0755	0.00114	0.0381	0.34	203298_s_at
131	2934	-2.01	0.0679	0.001149	0.0381	0.343	204012_s_at
132	2856	-2.01	0.1542	0.001158	0.0381	0.308	203888_at
133	5204	-2	0.145	0.001167	0.0381	0.308	209494_s_at
134	9815	2	0.1007	0.001175	0.0381	3.035	219228_at
135	9371	2	0.0776	0.001184	0.0381	2.929	218631_at
136	10221	-1.99	0.1474	0.001193	0.0381	0.313	220085_at
137	8568	1.98	0.0885	0.001202	0.0381	2.962	217741_s_at
138	5608	1.98	0.1706	0.001211	0.0381	3.346	210357_s_at
139	7972	-1.98	0.1182	0.001219	0.0381	0.326	215068_s_at
140	10758	-1.97	0.0803	0.001228	0.0381	0.345	221787_at
141	93	-1.97	0.1248	0.001237	0.0381	0.324	200050_at
142	1760	-1.97	0.1202	0.001246	0.0381	0.328	14 202421_at
143	10085	-1.97	0.1028	0.001254	0.0381	0.336	219737_s_at
144	6910	-1.97	0.0769	0.001263	0.0381	0.347	212888_at
145	6270	-1.97	0.0925	0.001272	0.0381	0.341	212098_at
146	9319	1.96	0.0946	0.001281	0.0381	2.946	218566_s_at
147	6343	-1.96	0.2213	0.001289	0.0381	0.282	212179_at
148	6789	-1.96	0.0195	0.001298	0.0381	0.377	212731_at
149	9056	-1.96	0.0608	0.001307	0.0381	0.357	218263_s_at
150	9990	-1.96	0.1974	0.001316	0.0381	0.301	219538_at
151	5655	1.94	0.0834	0.001325	0.0381	2.877	210513_s_at
152	5272	-1.94	0.0894	0.001333	0.0381	0.346	209603_at
153	9105	1.94	0.0887	0.001342	0.0381	2.9	218319_at
154	9260	-1.93	0.1135	0.001351	0.0381	0.337	218502_s_at
155	1556	-1.92	0.1238	0.00136	0.0381	0.335	202172_at
156	6240	-1.92	0.0383	0.001368	0.0381	0.374	212062_at
157	3227	-1.92	0.0912	0.001377	0.0381	0.348	204497_at
158	1644	-1.92	0.0662	0.001386	0.0381	0.362	202286_s_at
159	4785	-1.92	0.045	0.001395	0.0381	0.371	208954_s_at
160	9264	-1.92	0.143	0.001404	0.0381	0.326	218507_at
161	3616	-1.91	0.1448	0.001412	0.0381	0.327	205258_at
162	8503	-1.91	0.256	0.001421	0.0381	0.278	217523_at
163	313	-1.91	0.0469	0.00143	0.0381	0.372	200768_s_at
164	10012	-1.89	0.1933	0.001439	0.0381	0.31	219581_at
165	9431	-1.89	0.1724	0.001447	0.0381	0.323	218704_at
166	1122	-1.89	0.0859	0.001456	0.0381	0.358	201669_s_at
167	10680	1.89	0.0485	0.001465	0.0381	2.666	221667_s_at
168	6740	-1.88	0.1222	0.001474	0.0381	0.345	212660_at

169	1982	1.88	0.0335	0.001482	0.0381	2.598	202693_s_at
170	10165	-1.87	0.0741	0.001491	0.0381	0.366	219936_s_at
171	6926	-1.87	0.0698	0.0015	0.0381	0.368	212907_at
172	7411	-1.87	0.0995	0.001509	0.0381	0.355	213693_s_at
173	11296	-1.86	0.1215	0.001518	0.0381	0.348	53968_at
174	11028	-1.86	0.1635	0.001526	0.0381	0.332	222380_s_at
175	2386	-1.86	0.1819	0.001535	0.0381	0.317	203232_s_at
176	9052	-1.86	0.2075	0.001544	0.0381	0.306	218259_at
177	3409	-1.86	0.169	0.001553	0.0381	0.331	204827_s_at
178	7146	-1.84	0.1316	0.001561	0.0381	0.345	213229_at
179	3911	-1.84	0.107	0.00157	0.0381	0.358	206114_at
180	10204	-1.83	0.1625	0.001579	0.0381	0.332	220035_at
181	6285	-1.83	0.0845	0.001588	0.0381	0.37	212114_at
182	2645	1.83	0.0368	0.001596	0.0381	2.545	203574_at
183	5719	-1.83	0.205	0.001605	0.0381	0.322	210674_s_at
184	9971	-1.82	0.1982	0.001614	0.0381	0.324	219495_s_at
185	2302	-1.82	0.0843	0.001623	0.0381	0.372	203124_s_at
186	3897	-1.81	0.1217	0.001632	0.0381	0.356	206081_at
187	5027	-1.81	0.0809	0.00164	0.0381	0.374	209250_at
188	10441	-1.81	0.1468	0.001649	0.0381	0.349	221024_s_at
189	7151	-1.81	0.0813	0.001667	0.0381	0.377	213237_at
190	3510	-1.8	0.143	0.001675	0.0381	0.35	205034_at
191	4354	-1.79	0.1359	0.001702	0.0381	0.354	208042_at
192	8525	-1.79	0.123	0.001711	0.0381	0.36	217610_at
193	5878	-1.78	0.0982	0.001728	0.0381	0.373	211090_s_at
194	3117	-1.78	0.0658	0.001746	0.0381	0.389	204308_s_at
195	3376	-1.77	0.1035	0.001763	0.0381	0.374	204768_s_at
196	3375	-1.77	0.0911	0.001772	0.0381	0.379	204767_s_at
197	2196	-1.77	0.1214	0.001781	0.0381	0.368	202978_s_at
198	2939	-1.77	0.0437	0.001789	0.0381	0.403	204020_at
199	1849	-1.76	0.0429	0.001798	0.0381	0.403	202535_at
200	3323	-1.76	0.0342	0.001816	0.0381	0.408	204653_at
201	11367	-1.76	0.1335	0.001825	0.0381	0.362	65438_at
202	6992	-1.76	0.0451	0.001833	0.0381	0.404	213002_at
203	854	-1.76	0.2022	0.001842	0.0381	0.339	201367_s_at
204	8813	-1.75	0.0427	0.001851	0.0381	0.405	217997_at
205	9418	-1.75	0.1495	0.00186	0.0381	0.356	218689_at
206	7929	-1.75	0.1389	0.001868	0.0381	0.365	214948_s_at
207	7104	-1.75	0.2793	0.001877	0.0381	0.301	213153_at
208	3461	-1.75	0.3515	0.001886	0.0381	0.269	204932_at
209	1904	-1.74	0.1563	0.001895	0.0381	0.351	202599_s_at
210	2389	-1.74	0.0961	0.001904	0.0381	0.381	203238_s_at
211	9830	-1.74	0.1081	0.001912	0.0381	0.378	219253_at
212	2192	-1.74	0.0843	0.001921	0.0381	0.39	202969_at
213	9950	-1.73	0.0641	0.00193	0.0381	0.4	219459_at
214	2346	-1.73	0.1411	0.001939	0.0381	0.369	203188_at
215	6612	-1.73	0.1308	0.001947	0.0381	0.367	212504_at

Table S2. Differentially-expressed genes using the EBAM method. The raw (unadjusted) *p*-value and the fold-difference is given for each gene. Also given is the "d.value" (the modified *t*-test statistic) and its standard error.

Identified Genes (posterior >=0.7):

	Row	z.value	posterior	local.fdr	Name
1	11114	173.71	0.71	0.29004	36711_at
2	9356	104.14	0.9616	0.0384	218611_at
3	11125	87.89	0.9697	0.03025	37028_at
4	3208	73.97	0.9723	0.02771	204472_at
5	8426	71.01	0.9724	0.02762	217168_s_at
6	4842	59.07	0.971	0.02902	209020_at
7	4600	58.74	0.9709	0.0291	208744_x_at
8	1888	56.09	0.9701	0.02986	202581_at
9	936	55.75	0.97	0.02997	201464_x_at
10	9612	50.85	0.9681	0.03192	218936_s_at
11	6337	48.69	0.967	0.03302	212171_x_at
12	341	45.66	0.9651	0.03486	200800_s_at
13	2718	42.93	0.9632	0.03684	203665_at
14	1982	41.14	0.9616	0.0384	202693_s_at
15	5123	41.11	0.9616	0.03842	209383_at
16	9446	41.1	0.9616	0.03843	218723_s_at
17	213	40.68	0.9612	0.03884	200664_s_at
18	4637	40.45	0.9609	0.03907	208786_s_at
19	4641	37.36	0.9573	0.04273	208792_s_at
20	417	36.82	0.9565	0.04352	200880_at
21	2097	36.77	0.9564	0.0436	202842_s_at
22	2645	36.54	0.9561	0.04394	203574_at
23	6129	36.38	0.9558	0.04419	211939_x_at
24	887	36.07	0.9553	0.04469	201405_s_at
25	1535	35.6	0.9545	0.04548	202147_s_at
26	1146	33.64	0.9507	0.04927	201694_s_at
27	7556	33.6	0.9507	0.04935	213988_s_at
28	217	33.05	0.9494	0.0506	200669_s_at
29	4807	32.38	0.9478	0.05225	208980_s_at
30	1966	32.11	0.9471	0.05294	202672_s_at
31	4593	32.02	0.9468	0.05319	208737_at
32	340	31.96	0.9467	0.05333	200799_at
33	1421	30.94	0.9437	0.05627	202014_at
34	2888	30.87	0.9435	0.05648	203946_s_at
35	4259	30.74	0.9431	0.05689	207630_s_at
36	4364	30.57	0.9425	0.05746	208078_s_at
37	2759	30.47	0.9422	0.05778	203725_at
38	4102	30.41	0.942	0.05798	206976_s_at
39	7119	30.2	0.9413	0.05866	213187_x_at
40	5728	30.08	0.9409	0.05907	210691_s_at
41	6154	30.01	0.9407	0.05932	211968_s_at

42	348	29.79	0.9399	0.06009	200807_s_at
43	10680	29.16	0.9376	0.06244	221667_s_at
44	323	27.71	0.9313	0.06873	200779_at
45	8560	27.58	0.9307	0.06934	217733_s_at
46	7808	27.55	0.9305	0.06947	214683_s_at
47	418	27.23	0.9289	0.07108	200881_s_at
48	8608	27.17	0.9286	0.07142	217783_s_at
49	6781	27.15	0.9285	0.0715	212722_s_at
50	4548	27.13	0.9284	0.07159	208687_x_at
51	4658	26.98	0.9276	0.0724	208810_at
52	79	26.72	0.9262	0.07378	200036_s_at
53	3109	26.59	0.9255	0.07454	204285_s_at
54	4661	26.36	0.9242	0.07581	208813_at
55	6971	26.24	0.9235	0.07655	212971_at
56	937	26.05	0.9223	0.07767	201466_s_at
57	1325	25.38	0.9181	0.08192	201898_s_at
58	565	25.03	0.9157	0.08432	201041_s_at
59	214	24.87	0.9145	0.08546	200666_s_at
60	10219	24.84	0.9143	0.08566	220081_x_at
61	7657	24.58	0.9124	0.08759	214211_at
62	4636	24.57	0.9123	0.08771	208785_s_at
63	6061	24.54	0.9121	0.08792	211746_x_at
64	10795	24.43	0.9112	0.08879	221841_s_at
65	2875	24.37	0.9108	0.08924	203925_at
66	4659	24.15	0.9091	0.09094	208811_s_at
67	5684	24.04	0.9081	0.09189	210592_s_at
68	10373	23.71	0.9053	0.09467	220755_s_at
69	6179	23.33	0.902	0.098	211998_at
70	1372	22.96	0.8985	0.10153	201953_at
71	7717	22.74	0.8963	0.10367	214359_s_at
72	4465	22.71	0.896	0.10403	208540_x_at
73	2002	22.63	0.8952	0.10479	202720_at
74	9504	22.6	0.8949	0.10514	218794_s_at
75	432	22.26	0.8913	0.10868	200895_s_at
76	3303	22.21	0.8907	0.10931	204621_s_at
77	8420	22.19	0.8905	0.10947	217144_at
78	107	22.16	0.8902	0.10979	200064_at
79	8196	21.71	0.885	0.11499	215948_x_at
80	1534	21.67	0.8846	0.11541	202146_at
81	4275	21.54	0.8829	0.11709	207714_s_at
82	8267	21.51	0.8826	0.11742	216274_s_at
83	1825	21.49	0.8824	0.11765	202503_s_at
84	4117	21.39	0.8811	0.11887	207040_s_at
85	1659	21.34	0.8805	0.11954	202302_s_at
86	10625	21.27	0.8796	0.1204	221577_x_at
87	5654	21.24	0.8792	0.12076	210512_s_at
88	11172	21.24	0.8792	0.12082	39729_at

89	6148	21.14	0.8778	0.12215	211960_s_at
90	459	21.12	0.8776	0.1224	200924_s_at
91	1851	20.96	0.8754	0.12456	202537_s_at
92	5510	20.95	0.8753	0.12468	210101_x_at
93	6782	20.91	0.8748	0.12518	212723_at
94	330	20.89	0.8745	0.12552	200788_s_at
95	8559	20.74	0.8724	0.12757	217732_s_at
96	765	20.41	0.8677	0.13234	201263_at
97	2966	20.33	0.8665	0.13351	204059_s_at
98	127	20.31	0.8662	0.13384	200084_at
99	359	20.04	0.8621	0.13795	200818_at
100	9371	20.01	0.8615	0.13846	218631_at
101	10987	19.93	0.8604	0.13964	222239_s_at
102	1464	19.91	0.8599	0.14008	202068_s_at
103	6609	19.9	0.8599	0.14012	212501_at
104	7080	19.9	0.8598	0.14018	213124_at
105	37	19.84	0.8588	0.14122	121_at
106	1310	19.65	0.8556	0.14438	201881_s_at
107	2177	19.64	0.8554	0.14459	202949_s_at
108	997	19.6	0.8548	0.14519	201531_at
109	4565	19.54	0.8538	0.14618	208705_s_at
110	684	19.49	0.8528	0.14718	201173_x_at
111	5809	19.41	0.8515	0.14845	210950_s_at
112	1346	19.31	0.8498	0.15021	201926_s_at
113	176	19.3	0.8496	0.1504	200627_at
114	3304	19.19	0.8476	0.1524	204622_x_at
115	1853	19.16	0.847	0.15302	202539_s_at
116	7593	19.14	0.8465	0.15347	214073_at
117	4049	19.1	0.8459	0.15413	206744_s_at
118	5706	18.98	0.8435	0.15646	210648_x_at
119	3937	18.94	0.8428	0.15724	206247_at
120	1307	18.9	0.842	0.15802	201878_at
121	5840	18.72	0.8386	0.16142	211025_x_at
122	216	18.62	0.8365	0.16351	200668_s_at
123	5100	18.57	0.8354	0.16458	209345_s_at
124	5655	18.25	0.8288	0.17119	210513_s_at
125	3004	18.13	0.826	0.17397	204119_s_at
126	2085	18.07	0.8247	0.17533	202824_s_at
127	1039	18.05	0.8243	0.1757	201575_at
128	4567	18.05	0.8243	0.17573	208708_x_at
129	3058	18.02	0.8235	0.17647	204203_at
130	4757	17.72	0.8165	0.18349	208926_at
131	8568	17.66	0.8153	0.18475	217741_s_at
132	1196	17.66	0.8152	0.18483	201751_at
133	6743	17.5	0.8113	0.18874	212665_at
134	8893	17.42	0.8092	0.19078	218088_s_at
135	9011	17.4	0.8086	0.19138	218214_at

136	5833	17.37	0.8079	0.19207	211015_s_at
137	5540	17.37	0.8078	0.19215	210183_x_at
138	7732	17.35	0.8074	0.19259	214431_at
139	10599	17.33	0.8068	0.19321	221539_at
140	9105	17.26	0.8052	0.19478	218319_at
141	2916	17.24	0.8047	0.19531	203984_s_at
142	11373	17.12	0.8015	0.19845	65588_at
143	1069	17.01	0.7984	0.20165	201605_x_at
144	4114	16.93	0.7962	0.20383	207030_s_at
145	767	16.86	0.7942	0.20577	201266_at
146	448	16.84	0.7937	0.20628	200912_s_at
147	4970	16.76	0.7916	0.2084	209173_at
148	8793	16.72	0.7903	0.20967	217977_at
149	3828	16.53	0.7848	0.21518	205807_s_at
150	9319	16.49	0.7836	0.21644	218566_s_at
151	10246	16.36	0.7798	0.22022	220173_at
152	356	16.36	0.7798	0.22025	200815_s_at
153	4791	16.33	0.779	0.22105	208961_s_at
154	4414	16.23	0.7758	0.22422	208290_s_at
155	3110	16.18	0.7744	0.2256	204286_s_at
156	8336	16.18	0.7743	0.2257	216607_s_at
157	5180	16.13	0.7727	0.22733	209457_at
158	8671	16.1	0.7718	0.2282	217848_s_at
159	7594	16.1	0.7717	0.22826	214074_s_at
160	4633	16.09	0.7716	0.22843	208781_x_at
161	7330	16.04	0.7699	0.23014	213545_x_at
162	6126	16	0.7686	0.23138	211936_at
163	6396	16	0.7685	0.23148	212241_at
164	10266	15.99	0.7681	0.23188	220235_s_at
165	4837	15.97	0.7677	0.23226	209015_s_at
166	9815	15.93	0.7663	0.23368	219228_at
167	9329	15.93	0.7662	0.23383	218576_s_at
168	60	15.91	0.7656	0.23439	200017_at
169	5407	15.8	0.7621	0.23787	209882_at
170	6836	15.78	0.7614	0.23858	212788_x_at
171	6021	15.78	0.7613	0.23872	211662_s_at
172	3294	15.68	0.758	0.24203	204605_at
173	7606	15.65	0.7569	0.24315	214096_s_at
174	2098	15.48	0.7511	0.24888	202843_at
175	5661	15.4	0.7483	0.2517	210532_s_at
176	5080	15.28	0.744	0.25596	209318_x_at
177	3241	15.27	0.7435	0.25653	204517_at
178	6397	15.25	0.7431	0.25695	212242_at
179	2581	15.09	0.737	0.26304	203484_at
180	4880	15.07	0.7364	0.26359	209062_x_at
181	1509	15.05	0.7356	0.26439	202120_x_at
182	440	15.05	0.7355	0.26454	200903_s_at

183	5968	15.02	0.7344	0.26558	211475_s_at
184	10269	14.97	0.7326	0.26745	220238_s_at
185	110	14.96	0.732	0.268	200067_x_at
186	4359	14.9	0.7298	0.27021	208066_s_at
187	2563	14.86	0.7284	0.27159	203455_s_at
188	685	14.8	0.7262	0.27383	201174_s_at
189	66	14.78	0.7252	0.27481	200023_s_at
190	8270	14.75	0.7239	0.2761	216295_s_at
191	1778	14.74	0.7235	0.27649	202442_at
192	10496	14.71	0.7223	0.2777	221249_s_at
193	6007	14.68	0.7212	0.27882	211600_at
194	2781	14.65	0.7201	0.27989	203752_s_at
195	7815	14.61	0.7186	0.2814	214696_at
196	8988	14.6	0.7181	0.28191	218190_s_at
197	4983	14.58	0.7175	0.28254	209189_at
198	343	14.51	0.7146	0.28544	200802_at
199	1463	14.4	0.7101	0.2899	202067_s_at
200	470	14.35	0.7077	0.29234	200937_s_at
201	4950	14.32	0.7068	0.29322	209146_at
202	182	14.32	0.7067	0.29331	200633_at
203	1120	14.3	0.29427	0.29427	201666_at
204	10788	14.29	0.7053	0.29468	221827_at
205	651	14.29	0.7052	0.29476	201135_at
206	7746	14.26	0.29584	0.29584	214472_at
207	735	14.26	0.7039	0.29607	201229_s_at
208	3269	14.24	0.7032	0.29676	204566_at
209	2817	14.23	0.7026	0.29739	203810_at
210	1216	14.21	0.702	0.29799	201772_at
211	7887	14.21	0.702	0.29802	214827_at

Table S3. Genes in common in Tables S1 and S2.

1	200664_s_at
2	200666_s_at
3	200799_at
4	200800_s_at
5	200924_s_at
6	201041_s_at
7	201464_x_at
8	201466_s_at
9	201694_s_at
10	202014_at
11	202146_at
12	202147_s_at
13	202581_at
14	202672_s_at
15	202693_s_at
16	202842_s_at

17	202843_at
18	203455_s_at
19	203574_at
20	203665_at
21	203725_at
22	203810_at
23	203946_s_at
24	204285_s_at
25	204286_s_at
26	204472_at
27	204621_s_at
28	204622_x_at
29	206976_s_at
30	207030_s_at
31	207630_s_at
32	207714_s_at
33	208078_s_at
34	208744_x_at
35	208786_s_at
36	209020_at
37	209189_at
38	209383_at
39	210512_s_at
40	210513_s_at
41	210592_s_at
42	212171_x_at
43	212722_s_at
44	212723_at
45	213988_s_at
46	214683_s_at
47	214696_at
48	217144_at
49	217168_s_at
50	217741_s_at
51	218319_at
52	218566_s_at
53	218611_at
54	218631_at
55	218723_s_at
56	219228_at
57	221577_x_at
58	221667_s_at
59	36711_at
60	37028_at
61	65588_at

Table S4. The diterpene connectivity scores for the 453 "instances" (representing treatment of various cell lines with 164 different compounds).

Rank	Instance id	Cmap name	Batch	Dose	Cell line	Score	Up	Down	Commments
1	1069	15-delta prostaglandin J2	513	10 µM	MCF7	1	0.846	−0.651	PPAR gamma inhibitor
2	887	Celastrol	505	3 µM	MCF7	0.958	0.886	−0.548	proteasome inhibitor
3	1011	15-delta prostaglandin J2	506	10 µM	MCF7	0.957	0.8	−0.633	
4	1140	MG-132	514	21 µM	MCF7	0.866	0.711	−0.585	proteasome inhibitor
5	956	5224221	502	12 µM	MCF7	0.823	0.721	−0.511	
6	961	5253409	502	17 µM	MCF7	0.786	0.692	−0.485	
7	1068	Thioridazine	513	10 µM	MCF7	0.785	0.686	−0.489	calmodulin inhibitor
8	906	Calmidazolium	505	5 µM	MCF7	0.756	0.691	−0.44	calmodulin inhibitor
9	992	trichostatin A	506	100 nM	MCF7	0.721	0.494	−0.585	HDAC inhibitor
10	941	rottlerin	502	10 µM	MCF7	0.715	0.626	−0.445	PKC inhibitor
11	835	carbamazepine	504	100 nM	MCF7	0.713	0.665	−0.402	sodium channel inhibitor
12	1010	thioridazine	506	10 µM	MCF7	0.712	0.679	−0.387	
13	844	5253409	504	17 µM	MCF7	0.711	0.718	−0.347	
14	981	trichostatin A	502	1 µM	MCF7	0.693	0.545	−0.493	
15	839	5224221	504	12 µM	MCF7	0.692	0.673	−0.363	
16	1072	trichostatin A	513	1 µM	MCF7	0.69	0.558	−0.475	
17	1058	vorinostat	513	10 µM	MCF7	0.69	0.526	−0.507	
18	1014	trichostatin A	506	1 µM	MCF7	0.686	0.524	−0.503	
19	949	5255229	502	13 µM	MCF7	0.682	0.669	−0.352	
20	1000	vorinostat	506	10 µM	MCF7	0.679	0.516	−0.501	
21	909	HC toxin	505	100 nM	MCF7	0.676	0.571	−0.441	
22	831	17-allylamino-geldanamycin	504	1 µM	MCF7	0.667	0.586	−0.412	HSP90 inhibitor
23	952	carbamazepine	502	100 nM	MCF7	0.664	0.648	−0.346	
24	904	5109870	505	25 µM	MCF7	0.663	0.528	−0.465	
25	965	felodipine	502	10 µM	MCF7	0.661	0.745	−0.245	
26	1050	trichostatin A	513	100 nM	MCF7	0.655	0.443	−0.538	
27	1053	prochlorperazine	513	10 µM	MCF7	0.654	0.476	−0.503	
28	1004	trifluoperazine	506	10 µM	MCF7	0.653	0.519	−0.458	
29	947	17-allylamino-geldanamycin	502	1 µM	MCF7	0.648	0.557	−0.413	
30	873	trichostatin A	504	1 µM	MCF7	0.641	0.5	−0.459	
31	486	calmidazolium	67	5 µM	MCF7	0.639	0.666	−0.29	
32	976	5182598	502	25 µM	MCF7	0.633	0.579	−0.369	
33	1064	17-allylamino-geldanamycin	513	1 µM	MCF7	0.631	0.495	−0.449	
34	1066	geldanamycin	513	1 µM	MCF7	0.628	0.486	−0.454	
35	841	resveratrol	504	10 µM	MCF7	0.621	0.511	−0.418	
36	1056	17-allylamino-geldanamycin	513	1 µM	MCF7	0.615	0.462	−0.458	
37	971	oxaprozin	502	300 µM	MCF7	0.613	0.538	−0.38	
38	1112	trichostatin A	514	100 nM	MCF7	0.613	0.438	−0.479	
39	868	5182598	504	25 µM	MCF7	0.61	0.534	−0.379	
40	1051	17-dimethylamino-geldanamycin	513	100 nM	MCF7	0.609	0.457	−0.454	
41	848	felodipine	504	10 µM	MCF7	0.608	0.615	−0.295	
42	958	resveratrol	502	10 µM	MCF7	0.607	0.525	−0.384	

Table S4. *Cont.*

Rank	Instance id	Cmap name	Batch	Dose	Cell line	Score	Up	Down	Commments
43	1063	17-allylamino-geldanamycin	513	1 µM	MCF7	0.607	0.517	−0.391	
44	1075	fluphenazine	513	10 µM	MCF7	0.605	0.494	−0.411	
45	864	geldanamycin	504	1 µM	MCF7	0.595	0.595	−0.296	
46	910	trifluoperazine	505	10 µM	MCF7	0.594	0.508	−0.381	
47	870	pyrvinium	504	1 µM	MCF7	0.589	0.484	−0.397	
48	916	17-allylamino-geldanamycin	505	1 µM	MCF7	0.583	0.536	−0.337	
49	1044	17-allylamino-geldanamycin	513	1 µM	MCF7	0.582	0.399	−0.473	
50	998	17-allylamino-geldanamycin	506	1 µM	MCF7	0.58	0.524	−0.345	
51	995	prochlorperazine	506	10 µM	MCF7	0.576	0.48	−0.383	
52	1132	BW-B70C	514	32 µM	MCF7	0.573	0.546	−0.312	
53	332	trichostatin A	28	100 nM	MCF7	0.572	0.477	−0.379	
54	1005	17-allylamino-geldanamycin	506	1 µM	MCF7	0.571	0.434	−0.421	
55	1008	geldanamycin	506	1 µM	MCF7	0.57	0.355	−0.498	
56	986	17-allylamino-geldanamycin	506	1 µM	MCF7	0.568	0.432	−0.418	
57	972	geldanamycin	502	1 µM	MCF7	0.566	0.558	−0.289	
58	953	monorden	502	100 nM	MCF7	0.565	0.446	−0.4	
59	1057	monorden	513	100 nM	MCF7	0.561	0.488	−0.352	
60	1070	troglitazone	513	10 µM	MCF7	0.558	0.458	−0.377	
61	999	monorden	506	100 nM	MCF7	0.557	0.432	−0.402	
62	836	monorden	504	100 nM	MCF7	0.556	0.478	−0.355	
63	331	trichostatin A	28	100 nM	MCF7	0.553	0.354	−0.474	
64	993	17-dimethylamino-geldanamycin	506	100 nM	MCF7	0.548	0.372	−0.449	
65	881	docosahexaenoic acid ethyl ester	505	100 µM	MCF7	0.547	0.401	−0.418	
66	1006	17-allylamino-geldanamycin	506	1 µM	MCF7	0.531	0.434	−0.361	
67	978	pyrvinium	502	1 µM	MCF7	0.528	0.362	−0.428	
68	977	wortmannin	502	1 µM	MCF7	0.527	0.468	−0.321	
69	1114	tyrphostin AG-0	514	25 µM	MCF7	0.52	0.174	−0.605	
70	1017	fluphenazine	506	10 µM	MCF7	0.517	0.453	−0.321	
71	882	ionomycin	505	2 µM	MCF7	0.515	0.343	−0.428	
72	863	oxaprozin	504	300 µM	MCF7	0.512	0.504	−0.262	
73	1012	troglitazone	506	10 µM	MCF7	0.51	0.429	−0.334	
74	901	5114445	505	10 µM	MCF7	0.509	0.275	−0.487	
75	905	clotrimazole	505	50 µM	MCF7	0.502	0.46	−0.292	
76	413	trichostatin A	45	100 nM	ssMCF7	0.5	0.325	−0.424	
77	869	wortmannin	504	1 µM	MCF7	0.493	0.493	−0.245	
78	611	geldanamycin	101	1 µM	MCF7	0.491	0.364	−0.371	
79	1061	nordihydroguaiaretic acid	513	1 µM	MCF7	0.488	0.479	−0.251	
80	994	valproic acid	506	200 µM	MCF7	0.488	0.293	−0.438	
81	893	pararosaniline	505	10 µM	MCF7	0.487	0.393	−0.336	
82	914	rottlerin	505	10 µM	MCF7	0.478	0.362	−0.353	
83	521	17-allylamino-geldanamycin	74	1 µM	ssMCF7	0.478	0.328	−0.388	
84	489	monorden	68	100 nM	MCF7	0.477	0.375	−0.339	
85	345	valproic acid	33	10 mM	MCF7	0.472	0.345	−0.362	
86	1141	tyrphostin AG-0	514	32 µM	MCF7	0.467	0.466	−0.233	

Table S4. *Cont.*

Rank	Instance id	Cmap name	Batch	Dose	Cell line	Score	Up	Down	Commments
87	681	monastrol	117	100 μM	MCF7	0.465	0.36	−0.336	
88	903	5151277	505	14 μM	MCF7	0.462	0.427	−0.264	
89	612	LM-1685	101	10 μM	MCF7	0.46	0.33	−0.358	
90	979	ionomycin	502	2 μM	MCF7	0.459	0.414	−0.273	
91	607	butein	98	10 μM	MCF7	0.458	0.443	−0.242	
92	448	trichostatin A	60	100 nM	PC3	0.454	0.43	−0.25	
93	954	blebbistatin	502	17 μM	MCF7	0.446	0.245	−0.423	
94	837	blebbistatin	504	17 μM	MCF7	0.443	0.275	−0.388	
95	1048	alpha-estradiol	513	10 nM	MCF7	0.443	0.203	−0.46	
96	838	5248896	504	11 μM	MCF7	0.442	0.293	−0.368	
97	446	15-delta prostaglandin J2	60	10 μM	PC3	0.441	0.427	−0.233	
98	908	5140203	505	15 μM	MCF7	0.436	0.306	−0.347	
99	1041	haloperidol	513	10 μM	MCF7	0.434	0.265	−0.385	
100	622	resveratrol	107	50 μM	MCF7	0.433	0.271	−0.377	
101	505	17-allylamino-geldanamycin	73	1 μM	SKMEL5	0.427	0.423	−0.216	
102	381	17-allylamino-geldanamycin	40	1 μM	MCF7	0.422	0.388	−0.244	
103	825	rottlerin	504	10 μM	MCF7	0.422	0.264	−0.367	
104	913	colforsin	505	50 μM	MCF7	0.421	0.32	−0.31	
105	966	tretinoin	502	1 μM	MCF7	0.417	0.216	−0.408	
106	334	mercaptopurine	28	100 μM	MCF7	0.416	0.281	−0.342	
107	1015	genistein	506	10 μM	MCF7	0.414	0.187	−0.433	
108	889	5286656	505	50 μM	MCF7	0.412	0.287	−0.33	
109	593	geldanamycin	95	1 μM	MCF7	0.411	0.402	−0.214	
110	595	resveratrol	95	50 μM	MCF7	0.411	0.33	−0.285	
111	1047	valproic acid	513	1 mM	MCF7	0.408	0.246	−0.365	
112	346	valproic acid	33	2 mM	MCF7	0.407	0.305	−0.304	
113	989	valproic acid	506	1 mM	MCF7	0.401	0.25	−0.35	
114	423	staurosporine	53	100 nM	MCF7	0.399	0.199	−0.398	
115	1055	chlorpromazine	513	1 μM	MCF7	0.397	0.186	−0.408	
116	944	5252917	502	14 μM	MCF7	0.394	0.284	−0.306	
117	1020	valproic acid	506	500 μM	MCF7	0.392	0.322	−0.265	
118	950	5211181	502	12 μM	MCF7	0.392	0.218	−0.369	
119	1049	tretinoin	513	1 μM	MCF7	0.392	0.179	−0.408	
120	892	5162773	505	7 μM	MCF7	0.387	0.292	−0.288	
121	988	estradiol	506	100 nM	MCF7	0.386	0.304	−0.274	
122	428	17-allylamino-geldanamycin	54	1 μM	MCF7	0.383	0.342	−0.231	
123	1076	fulvestrant	513	10 nM	MCF7	0.376	0.241	−0.322	
124	1065	LY-294002	513	10 μM	MCF7	0.375	0.228	−0.333	
125	1042	acetylsalicylic acid	513	100 μM	MCF7	0.375	0.16	−0.402	
126	432	17-allylamino-geldanamycin	55	1 μM	PC3	0.374	0.349	−0.211	
127	664	docosahexaenoic acid ethyl ester	90	100 μM	PC3	0.371	0.398	−0.158	
128	983	haloperidol	506	10 μM	MCF7	0.37	0.233	−0.321	
129	898	5213008	505	18 μM	MCF7	0.369	0.302	−0.25	
130	871	ionomycin	504	2 μM	MCF7	0.369	0.285	−0.268	

Table S4. *Cont.*

Rank	Instance id	Cmap name	Batch	Dose	Cell line	Score	Up	Down	Commments
131	842	bucladesine	504	2 µM	MCF7	0.369	0.267	−0.285	
132	630	colchicine	109	1 µM	SKMEL5	0.367	0.383	−0.166	
133	582	butein	87	10 µM	PC3	0.365	0.333	−0.214	
134	1003	nordihydroguaiaretic acid	506	1 µM	MCF7	0.36	0.254	−0.285	
135	431	troglitazone	55	10 µM	PC3	0.359	0.27	−0.268	
136	843	5279552	504	22 µM	MCF7	0.359	0.193	−0.345	
137	639	pentamidine	111	100 µM	MCF7	0.358	0.247	−0.289	
138	610	monastrol	101	100 µM	MCF7	0.357	0.196	−0.339	
139	584	dimethyloxalylglycine	87	1 mM	PC3	0.354	0.246	−0.284	
140	955	5248896	502	11 µM	MCF7	0.351	0.198	−0.328	
141	410	valproic acid	44	10 mM	HL60	0.347	0.342	−0.178	
142	849	tretinoin	504	1 µM	MCF7	0.345	0.264	−0.253	
143	1009	clozapine	506	10 µM	MCF7	0.343	0.197	−0.317	
144	201	15-delta prostaglandin J2	13	10 µM	MCF7	0.342	0.336	−0.176	
145	1024	haloperidol	506	10 µM	MCF7	0.337	0.254	−0.25	
146	497	valproic acid	70	1 mM	ssMCF7	0.333	0.237	−0.261	
147	417	thioridazine	46	10 µM	MCF7	0.327	0.265	−0.225	
148	455	prochlorperazine	62	10 µM	MCF7	0.325	0.247	−0.239	
149	421	trifluoperazine	53	10 µM	MCF7	0.323	0.297	−0.186	
150	609	5666823	101	100 µM	MCF7	0.323	0.263	−0.221	
151	338	azathioprine	29	100 µM	MCF7	0.318	0.239	−0.237	
152	874	depudecin	504	1 µM	MCF7	0.315	0.24	−0.232	
153	942	prazosin	502	10 µM	MCF7	0.31	0.218	−0.246	
154	662	resveratrol	90	50 µM	PC3	0.309	0.267	−0.196	
155	481	pirinixic acid	66	100 µM	PC3	0.307	0.278	−0.182	
156	123	dexamethasone	5	1 µM	MCF7	0.307	0.161	−0.298	
157	583	HNMPA-(AM)3	87	5 µM	PC3	0.306	0.248	−0.21	
158	1043	fulvestrant	513	1 µM	MCF7	0.303	0.171	−0.283	
159	590	3-aminobenzamide	94	10 mM	MCF7	0.299	0.183	−0.264	
160	573	deferoxamine	82	100 µM	MCF7	0.296	0.22	−0.223	
161	917	quercetin	505	1 µM	MCF7	0.295	0.221	−0.22	
162	866	ikarugamycin	504	2 µM	MCF7	0.292	0.229	−0.208	
163	629	valproic acid	109	1 mM	SKMEL5	0.291	0.263	−0.172	
164	970	5230742	502	17 µM	MCF7	0.287	0.208	−0.222	
165	430	rosiglitazone	55	10 µM	PC3	0.286	0.294	−0.134	
166	344	2-deoxy-D-glucose	31	10 mM	MCF7	0.285	0.273	−0.153	
167	621	nocodazole	107	1 µM	MCF7	0.285	0.173	−0.253	
168	308	sulindac sulfide	23	50 µM	MCF7	0.283	0.223	−0.2	
169	603	nifedipine	96	10 µM	MCF7	0.282	0.148	−0.274	
170	832	Y-27632	504	3 µM	MCF7	0.279	0.18	−0.237	
171	575	copper sulfate	82	100 µM	MCF7	0.278	0.267	−0.149	
172	141	chlorpropamide	6	100 µM	MCF7	0.275	0.144	−0.268	
173	333	diclofenac	28	10 µM	MCF7	0.274	0.146	−0.264	
174	347	valproic acid	33	500 µM	MCF7	0.273	0.158	−0.25	

Table S4. *Cont.*

Rank	Instance id	Cmap name	Batch	Dose	Cell line	Score	Up	Down	Commments
175	415	nordihydroguaiaretic acid	45	1 µM	ssMCF7	0.272	0.117	−0.29	
176	704	fulvestrant	110b	1 µM	PC3	0.271	0.229	−0.177	
177	642	tioguanine	112	10 µM	MCF7	0.267	0.141	−0.258	
178	667	mercaptopurine	116	10 µM	PC3	0.263	0.142	−0.252	
179	384	tretinoin	40	1 µM	MCF7	0.262	0.086	−0.306	
180	221	17-allylamino-geldanamycin	17	100 nM	MCF7	0.26	0.255	−0.134	
181	452	indometacin	61	100 µM	PC3	0.257	0.258	−0.126	
182	632	novobiocin	109	100 µM	SKMEL5	0.253	0.196	−0.182	
183	668	monastrol	116	100 µM	PC3	0.253	0.109	−0.27	
184	261	ciclosporin	20	1 µM	MCF7	0.251	0.125	−0.251	
185	638	genistein	111	10 µM	MCF7	0.248	0.235	−0.137	
186	168	sulindac	8	100 µM	MCF7	0.246	0.185	−0.183	
187	202	raloxifene	13	100 nM	MCF7	0.246	0.159	−0.21	
188	439	oxamic acid	58	10 mM	MCF7	0.244	0.108	−0.257	
189	23	valproic acid	2	1 mM	MCF7	0.242	0.183	−0.179	
190	433	valproic acid	56	1 mM	PC3	0.242	0.182	−0.181	
191	382	genistein	40	10 µM	MCF7	0.239	0.208	−0.15	
192	366	imatinib	36	10 µM	MCF7	0.237	0.218	−0.137	
193	440	W-13	58	10 µM	MCF7	0.234	0.091	−0.26	
194	490	fluphenazine	68	10 µM	MCF7	0.234	0.087	−0.263	
195	425	staurosporine	54	10 nM	MCF7	0.232	0.192	−0.156	
196	458	valproic acid	63	1 mM	PC3	0.232	0.185	−0.162	
197	631	benserazide	109	10 µM	SKMEL5	0.23	0.196	−0.148	
198	310	fulvestrant	23	10 nM	MCF7	0.221	0.164	−0.167	
199	464	pirinixic acid	65	100 µM	PC3	0.216	0.206	−0.117	
200	454	cobalt chloride	62	100 µM	MCF7	0.216	0.151	−0.172	
201	627	monastrol	108	100 µM	MCF7	0.214	0.131	−0.19	
202	422	thioridazine	53	1 µM	MCF7	0.213	0.183	−0.136	
203	348	valproic acid	33	50 µM	MCF7	0.21	0.187	−0.128	
204	205	rofecoxib	16	10 µM	MCF7	0.21	0.15	−0.164	
205	342	novobiocin	31	100 µM	MCF7	0.21	0.117	−0.197	
206	461	LY-294002	65	10 µM	PC3	0.207	0.181	−0.129	
207	317	N-phenylanthranilic acid	25	10 µM	MCF7	0.206	0.126	−0.182	
208	377	celecoxib	39	10 µM	MCF7	0.204	0.187	−0.118	
209	485	deferoxamine	67	100 µM	MCF7	0.203	0.146	−0.158	
210	666	butirosin	116	10 µM	PC3	0.202	0.144	−0.159	
211	203	nordihydroguaiaretic acid	13	1 µM	MCF7	0.202	0.119	−0.184	
212	1	metformin	1	10 µM	MCF7	0.201	0.107	−0.194	
213	501	LY-294002	71	10 µM	SKMEL5	0.2	0.194	−0.106	
214	252	celecoxib	18	10 µM	MCF7	0.2	0.121	−0.179	
215	416	clozapine	46	10 µM	MCF7	0.199	0.137	−0.161	
216	447	tretinoin	60	1 µM	PC3	0.196	0.12	−0.174	
217	702	alpha-estradiol	110b	10 nM	PC3	0.194	0.19	−0.101	
218	460	deferoxamine	63	100 µM	PC3	0.194	0.148	−0.143	

Table S4. *Cont.*

Rank	Instance id	Cmap name	Batch	Dose	Cell line	Score	Up	Down	Commments
219	424	LY-294002	53	100 nM	MCF7	0.194	0.145	−0.146	
220	251	rofecoxib	18	10 µM	MCF7	0.193	0.112	−0.177	
221	309	exisulind	23	50 µM	MCF7	0.191	0.141	−0.145	
222	703	genistein	110b	10 µM	PC3	0.19	0.192	−0.093	
223	311	monastrol	24	100 µM	MCF7	0.188	0.146	−0.136	
224	663	U0125	90	1 µM	PC3	0.188	0.126	−0.155	
225	207	LM-1685	16	10 µM	MCF7	0.188	0.118	−0.164	
226	264	MK-886	20	1 µM	MCF7	0.186	0.065	−0.213	
227	368	pirinixic acid	36	100 µM	MCF7	0.182	0.13	−0.142	
228	369	rosiglitazone	37	10 µM	HL60	0.18	0.101	−0.169	
229	375	tamoxifen	38	1 µM	ssMCF7	0.178	0.127	−0.139	
230	614	monastrol	103	20 µM	MCF7	0.175	0.114	−0.148	
231	592	probucol	94	10 µM	MCF7	0.166	0.094	−0.154	
232	541	gefitinib	75	10 µM	HL60	0.152	0.127	−0.101	
233	918	ikarugamycin	505	2 µM	MCF7	0	0.456	0.194	
234	450	17-allylamino-geldanamycin	61	1 µM	PC3	0	0.448	0.144	
235	922	celecoxib	505	10 µM	MCF7	0	0.405	0.283	
236	974	ikarugamycin	502	2 µM	MCF7	0	0.398	0.321	
237	1073	genistein	513	10 µM	MCF7	0	0.367	0.251	
238	920	decitabine	505	100 nM	MCF7	0	0.364	0.281	
239	960	5279552	502	22 µM	MCF7	0	0.353	0.219	
240	890	5149715	505	10 µM	MCF7	0	0.353	0.241	
241	493	monorden	69	100 nM	SKMEL5	0	0.352	0.134	
242	1082	haloperidol	513	10 µM	MCF7	0	0.339	0.378	
243	449	monorden	61	100 nM	PC3	0	0.338	0.199	
244	834	5211181	504	12 µM	MCF7	0	0.337	0.202	
245	265	prednisolone	21	1 µM	MCF7	0	0.336	0.283	
246	900	5186324	505	2 µM	MCF7	0	0.33	0.353	
247	325	monorden	26b	100 nM	MCF7	0	0.319	0.189	
248	982	depudecin	502	1 µM	MCF7	0	0.306	0.263	
249	1021	estradiol	506	10 nM	MCF7	0	0.299	0.324	
250	482	celecoxib	66	10 µM	PC3	0	0.288	0.212	
251	500	copper sulfate	70	100 µM	ssMCF7	0	0.286	0.199	
252	885	5186223	505	12 µM	MCF7	0	0.275	0.358	
253	327	arachidonyltrifluoromethane	26b	10 µM	MCF7	0	0.275	0.14	
254	407	sodium phenylbutyrate	43	1 mM	MCF7	0	0.272	0.276	
255	1103	demecolcine	514	12 µM	MCF7	0	0.27	0.246	
256	915	topiramate	505	3 µM	MCF7	0	0.27	0.359	
257	409	valproic acid	44	1 mM	HL60	0	0.263	0.118	
258	484	monorden	66	100 nM	PC3	0	0.261	0.107	
259	1105	monensin	514	11 µM	MCF7	0	0.26	0.236	
260	408	sodium phenylbutyrate	43	1 mM	MCF7	0	0.249	0.166	
261	438	copper sulfate	58	100 µM	MCF7	0	0.247	0.273	
262	488	iloprost	67	1 µM	MCF7	0	0.242	0.335	

Table S4. *Cont.*

Rank	Instance id	Cmap name	Batch	Dose	Cell line	Score	Up	Down	Commments
263	496	iloprost	69	1 µM	SKMEL5	0	0.238	0.094	
264	373	estradiol	38	10 nM	ssMCF7	0	0.232	0.214	
265	268	genistein	21	1 µM	MCF7	0	0.228	0.323	
266	437	novobiocin	58	100 µM	MCF7	0	0.227	0.156	
267	1138	phentolamine	514	12 µM	MCF7	0	0.226	0.298	
268	379	cobalt chloride	39	100 µM	MCF7	0	0.223	0.069	
269	1101	(-)-catechin	514	11 µM	MCF7	0	0.221	0.261	
270	542	SC-58125	75	10 µM	HL60	0	0.22	0.131	
271	564	15-delta prostaglandin J2	79	10 µM	SKMEL5	0	0.22	0.096	
272	1078	valproic acid	513	500 µM	MCF7	0	0.22	0.256	
273	1079	estradiol	513	10 nM	MCF7	0	0.22	0.23	
274	462	troglitazone	65	10 µM	PC3	0	0.214	0.122	
275	22	phenyl biguanide	2	10 µM	MCF7	0	0.209	0.22	
276	1121	DL-PPMP	514	2 µM	MCF7	0	0.206	0.323	
277	267	genistein	21	1 µM	MCF7	0	0.196	0.115	
278	1071	rosiglitazone	513	10 µM	MCF7	0	0.194	0.345	
279	594	arachidonyltrifluoromethane	95	10 µM	MCF7	0	0.193	0.201	
280	919	carbamazepine	505	100 nM	MCF7	0	0.192	0.245	
281	169	tacrolimus	8	1 µM	MCF7	0	0.189	0.16	
282	1060	valproic acid	513	50 µM	MCF7	0	0.187	0.223	
283	544	monorden	75	100 nM	HL60	0	0.186	0.062	
284	503	indometacin	71	100 µM	SKMEL5	0	0.186	0.091	
285	451	TTNPB	61	100 nM	PC3	0	0.178	0.188	
286	281	fludrocortisone	22a	1 µM	MCF7	0	0.175	0.163	
287	4	metformin	1	1 mM	MCF7	0	0.175	0.15	
288	256	rofecoxib	19	10 µM	MCF7	0	0.174	0.223	
289	142	tolbutamide	6	100 µM	MCF7	0	0.172	0.178	
290	374	dexamethasone	38	1 µM	ssMCF7	0	0.17	0.288	
291	388	raloxifene	41	100 nM	HL60	0	0.167	0.167	
292	492	haloperidol	68	10 µM	MCF7	0	0.166	0.128	
293	483	imatinib	66	10 µM	PC3	0	0.166	0.211	
294	383	cobalt chloride	40	100 µM	MCF7	0	0.162	0.201	
295	376	raloxifene	38	100 nM	ssMCF7	0	0.16	0.279	
296	1108	12,13-EODE	514	200 nM	MCF7	0	0.158	0.275	
297	222	tomelukast	17	1 µM	MCF7	0	0.156	0.202	
298	543	1,5-isoquinolinediol	75	100 µM	HL60	0	0.148	0.071	
299	284	tacrolimus	22a	1 µM	MCF7	0	0.146	0.244	
300	457	tetraethylenepentamine	63	100 µM	PC3	0	0.144	0.128	
301	307	sulindac	23	50 µM	MCF7	0	0.141	0.195	
302	426	chlorpromazine	54	1 µM	MCF7	0	0.135	0.173	
303	144	chlorpropamide	6	100 µM	MCF7	0	0.133	0.204	
304	266	thalidomide	21	100 µM	MCF7	0	0.132	0.256	
305	341	sodium phenylbutyrate	31	100 µM	MCF7	0	0.124	0.178	
306	387	estradiol	41	10 nM	HL60	0	0.121	0.139	

Table S4. *Cont.*

Rank	Instance id	Cmap name	Batch	Dose	Cell line	Score	Up	Down	Commments
307	343	fasudil	31	10 µM	MCF7	0	0.119	0.249	
308	782	estradiol	120	10 nM	HL60	0	0.115	0.098	
309	405	tetraethylenepentamine	43	10 µM	MCF7	0	0.107	0.199	
310	314	exisulind	24	50 µM	MCF7	0	0.104	0.137	
311	21	phenformin	2	10 µM	MCF7	0	0.091	0.122	
312	378	tacrolimus	39	1 µM	MCF7	0	0.091	0.08	
313	641	benserazide	112	10 µM	MCF7	0	0.089	0.255	
314	390	tretinoin	41	1 µM	HL60	0	0.089	0.111	
315	453	indometacin	62	100 µM	MCF7	0	−0.085	−0.227	
316	316	flufenamic acid	25	10 µM	MCF7	0	−0.086	−0.158	
317	605	monastrol	98	20 µM	MCF7	0	−0.09	−0.119	
318	576	novobiocin	82	100 µM	MCF7	0	−0.095	−0.286	
319	262	indometacin	20	20 µM	MCF7	0	−0.095	−0.258	
320	606	thalidomide	98	100 µM	MCF7	0	−0.096	−0.132	
321	370	troglitazone	37	10 µM	HL60	0	−0.098	−0.167	
322	204	sulfasalazine	13	100 µM	MCF7	0	−0.102	−0.224	
323	335	nifedipine	29	10 µM	MCF7	0	−0.104	−0.324	
324	365	estradiol	36	100 nM	MCF7	0	−0.104	−0.182	
325	337	felodipine	29	10 µM	MCF7	0	−0.106	−0.219	
326	419	chlorpromazine	46	10 µM	MCF7	0	−0.108	−0.284	
327	371	rofecoxib	37	10 µM	HL60	0	−0.108	−0.17	
328	367	fulvestrant	36	1 µM	MCF7	0	−0.109	−0.31	
329	602	ciclosporin	96	1 µM	MCF7	0	−0.111	−0.361	
330	143	tamoxifen	6	1 µM	MCF7	0	−0.111	−0.159	
331	223	TTNPB	17	100 nM	MCF7	0	−0.115	−0.265	
332	596	monastrol	95	100 µM	MCF7	0	−0.117	−0.19	
333	167	amitriptyline	8	1 µM	MCF7	0	−0.117	−0.131	
334	122	alpha-estradiol	5	10 nM	MCF7	0	−0.12	−0.294	
335	418	haloperidol	46	10 µM	MCF7	0	−0.125	−0.275	
336	361	LY-294002	35	10 µM	HL60	0	−0.126	−0.098	
337	412	tetraethylenepentamine	44	100 µM	HL60	0	−0.127	−0.113	
338	206	celecoxib	16	10 µM	MCF7	0	−0.128	−0.166	
339	121	estradiol	5	10 nM	MCF7	0	−0.129	−0.228	
340	165	exemestane	7	10 nM	MCF7	0	−0.132	−0.308	
341	665	estradiol	116	10 nM	PC3	0	−0.133	−0.224	
342	498	tetraethylenepentamine	70	100 µM	ssMCF7	0	−0.135	−0.218	
343	313	NU-1025	24	100 µM	MCF7	0	−0.135	−0.171	
344	414	estradiol	45	10 nM	ssMCF7	0	−0.139	−0.222	
345	224	tretinoin	17	1 µM	MCF7	0	−0.142	−0.316	
346	435	novobiocin	56	100 µM	PC3	0	−0.142	−0.189	
347	208	SC-58125	16	10 µM	MCF7	0	−0.143	−0.22	
348	1045	sirolimus	513	100 nM	MCF7	0	−0.148	−0.367	
349	436	fasudil	56	10 µM	PC3	0	−0.148	−0.267	
350	263	clofibrate	20	150 µM	MCF7	0	−0.149	−0.322	

Table S4. *Cont.*

Rank	Instance id	Cmap name	Batch	Dose	Cell line	Score	Up	Down	Commments
351	363	sodium phenylbutyrate	35	100 µM	HL60	0	−0.15	−0.083	
352	166	rofecoxib	7	10 µM	MCF7	0	−0.151	−0.308	
353	3	metformin	1	100 nM	MCF7	0	−0.151	−0.169	
354	495	pirinixic acid	69	100 µM	SKMEL5	0	−0.151	−0.123	
355	640	paclitaxel	111	100 nM	MCF7	0	−0.153	−0.183	
356	402	sirolimus	42	100 nM	ssMCF7	0	−0.154	−0.297	
357	434	sodium phenylbutyrate	56	1 mM	PC3	0	−0.155	−0.133	
358	601	MK-886	96	1 µM	MCF7	0	−0.156	−0.192	
359	401	LY-294002	42	10 µM	ssMCF7	0	−0.156	−0.276	
360	608	NU-1025	98	100 µM	MCF7	0	−0.156	−0.175	
361	523	fulvestrant	74	1 µM	ssMCF7	0	−0.16	−0.369	
362	1115	phenanthridinone	514	51 µM	MCF7	0	−0.16	−0.249	
363	364	trichostatin A	35	100 nM	HL60	0	−0.163	−0.081	
364	507	SC-58125	73	10 µM	SKMEL5	0	−0.163	−0.149	
365	487	pirinixic acid	67	100 µM	MCF7	0	−0.163	−0.342	
366	604	arachidonic acid	96	10 µM	MCF7	0	−0.164	−0.242	
367	336	nitrendipine	29	10 µM	MCF7	0	−0.164	−0.283	
368	318	LY-294002	25	10 µM	MCF7	0	−0.165	−0.278	
369	833	5255229	504	13 µM	MCF7	0	−0.166	−0.273	
370	404	wortmannin	42	10 nM	ssMCF7	0	−0.166	−0.409	
371	161	verapamil	7a	10 µM	MCF7	0	−0.172	−0.256	
372	315	acetylsalicylic acid	25	100 µM	MCF7	0	−0.173	−0.333	
373	494	fluphenazine	69	10 µM	SKMEL5	0	−0.173	−0.149	
374	124	mesalazine	5	100 µM	MCF7	0	−0.174	−0.355	
375	403	alpha-estradiol	42	10 nM	ssMCF7	0	−0.174	−0.358	
376	253	LM-1685	18	10 µM	MCF7	0	−0.174	−0.338	
377	491	dopamine	68	1 µM	MCF7	0	−0.177	−0.191	
378	1054	LY-294002	513	100 nM	MCF7	0	−0.178	−0.424	
379	380	tamoxifen	39	1 µM	MCF7	0	−0.179	−0.075	
380	255	dexamethasone	19	1 µM	MCF7	0	−0.18	−0.253	
381	624	4,5-dianilinophthalimide	107	10 µM	MCF7	0	−0.184	−0.321	
382	1122	cytochalasin B	514	21 µM	MCF7	0	−0.185	−0.267	
383	508	staurosporine	73	10 nM	SKMEL5	0	−0.185	−0.199	
384	524	nordihydroguaiaretic acid	74	1 µM	ssMCF7	0	−0.186	−0.347	
385	1113	doxycycline	514	14 µM	MCF7	0	−0.189	−0.271	
386	362	sirolimus	35	100 nM	HL60	0	−0.191	−0.119	
387	164	dexverapamil	7	10 µM	MCF7	0	−0.191	−0.321	
388	504	troglitazone	71	10 µM	SKMEL5	0	−0.191	−0.144	
389	258	LY-294002	19	10 µM	MCF7	0	−0.194	−0.238	
390	254	SC-58125	18	10 µM	MCF7	0	−0.195	−0.236	
391	1109	3-hydroxy-DL-kynurenine	514	9 µM	MCF7	0	−0.197	−0.292	
392	429	LY-294002	55	10 µM	PC3	0	−0.2	−0.206	
393	1080	sirolimus	513	100 nM	MCF7	0	−0.202	−0.305	
394	459	copper sulfate	63	100 µM	PC3	0	−0.208	−0.108	

Table S4. *Cont.*

Rank	Instance id	Cmap name	Batch	Dose	Cell line	Score	Up	Down	Commments
395	445	diclofenac	60	10 µM	PC3	0	−0.209	−0.181	
396	1059	sirolimus	513	100 nM	MCF7	0	−0.209	−0.304	
397	948	Y-27632	502	3 µM	MCF7	0	−0.214	−0.252	
398	502	sodium phenylbutyrate	71	200 µM	SKMEL5	0	−0.214	−0.118	
399	1074	LY-294002	513	10 µM	MCF7	0	−0.217	−0.317	
400	862	5230742	504	17 µM	MCF7	0	−0.22	−0.213	
401	985	fulvestrant	506	1 µM	MCF7	0	−0.22	−0.282	
402	1077	LY-294002	513	10 µM	MCF7	0	−0.244	−0.327	
403	896	5152487	505	10 µM	MCF7	0	−0.244	−0.375	
404	826	prazosin	504	10 µM	MCF7	0	−0.248	−0.332	
405	959	bucladesine	502	2 µM	MCF7	0	−0.25	−0.346	
406	1002	valproic acid	506	50 µM	MCF7	0	−0.251	−0.28	
407	312	staurosporine	24	1 µM	MCF7	0	−0.252	−0.575	
408	574	tetraethylenepentamine	82	100 µM	MCF7	0	−0.255	−0.176	
409	443	arachidonic acid	59	10 µM	MCF7	0	−0.258	−0.221	
410	984	acetylsalicylic acid	506	100 µM	MCF7	0	−0.261	−0.332	
411	991	tretinoin	506	1 µM	MCF7	0	−0.261	−0.344	
412	2	metformin	1	10 µM	MCF7	0	−0.265	−0.327	
413	828	5252917	504	14 µM	MCF7	0	−0.267	−0.283	
414	990	alpha-estradiol	506	10 nM	MCF7	0	−0.269	−0.349	
415	1007	LY-294002	506	10 µM	MCF7	0	−0.275	−0.403	
416	1019	LY-294002	506	10 µM	MCF7	0	−0.276	−0.281	
417	997	chlorpromazine	506	1 µM	MCF7	0	−0.305	−0.449	
418	1016	LY-294002	506	10 µM	MCF7	0	−0.317	−0.312	
419	1013	rosiglitazone	506	10 µM	MCF7	0	−0.318	−0.413	
420	506	wortmannin	73	10 nM	SKMEL5	0	−0.319	−0.211	
421	1001	sirolimus	506	100 nM	MCF7	0	−0.349	−0.337	
422	987	sirolimus	506	100 nM	MCF7	0	−0.36	−0.34	
423	579	fisetin	86	50 µM	PC3	0	−0.394	−0.231	
424	389	wortmannin	41	10 nM	HL60	−0.294	−0.097	0.09	
425	783	colforsin	120	50 µM	HL60	−0.309	−0.105	0.091	
426	61	metformin	2a	10 µM	MCF7	−0.334	−0.077	0.135	
427	282	fludrocortisone	22a	1 µM	MCF7	−0.334	−0.091	0.121	
428	283	quercetin	22a	1 µM	MCF7	−0.364	−0.106	0.125	
429	456	quinpirole	62	1 µM	MCF7	−0.406	−0.072	0.186	
430	643	W-13	112	10 µM	MCF7	−0.406	−0.108	0.15	
431	463	rofecoxib	65	10 µM	PC3	−0.406	−0.13	0.128	
432	411	sodium phenylbutyrate	44	1 mM	HL60	−0.433	−0.096	0.179	
433	661	splitomicin	90	20 µM	PC3	−0.435	−0.167	0.109	
434	444	clofibrate	59	100 µM	MCF7	−0.438	−0.111	0.167	
435	591	bucladesine	94	20 µM	MCF7	−0.438	−0.122	0.156	
436	427	iloprost	54	1 µM	MCF7	−0.479	−0.117	0.187	
437	644	colchicine	112	100 nM	MCF7	−0.488	−0.098	0.212	
438	442	oligomycin	59	1 µM	MCF7	−0.488	−0.133	0.177	

Table S4. *Cont.*

Rank	Instance id	Cmap name	Batch	Dose	Cell line	Score	Up	Down	Commments
439	406	tetraethylenepentamine	43	100 μM	MCF7	−0.506	−0.094	0.227	protease inhibitor
440	762	alpha-estradiol	119	10 nM	MCF7	−0.509	−0.18	0.143	
441	499	novobiocin	70	100 μM	ssMCF7	−0.523	−0.17	0.162	
442	578	4,5-dianilinophthalimide	86	10 μM	PC3	−0.535	−0.259	0.081	
443	441	arachidonic acid	59	10 μM	MCF7	−0.622	−0.17	0.225	
444	328	LY-294002	26b	10 μM	MCF7	−0.731	−0.257	0.207	
445	326	sirolimus	26b	100 nM	MCF7	−0.739	−0.163	0.306	
446	1135	minocycline	514	11 μM	MCF7	−0.776	−0.112	0.381	
447	1119	yohimbine	514	23 μM	MCF7	−0.776	−0.209	0.284	serotonin receptor antagonist
448	911	wortmannin	505	10 nM	MCF7	−0.847	−0.249	0.289	
449	1081	wortmannin	513	10 nM	MCF7	−0.866	−0.243	0.307	
450	921	sirolimus	505	100 nM	MCF7	−0.882	−0.242	0.318	
451	996	LY-294002	506	100 nM	MCF7	−0.894	−0.189	0.379	PI3 kinase inhibitor
452	1022	sirolimus	506	100 nM	MCF7	−0.915	−0.323	0.258	mTOR inhibitor
453	1023	wortmannin	506	10 nM	MCF7	−1	−0.368	0.267	PI3 kinase inhibitor

Table S5. Diterpene signature 3 fold annotated data.

Row	D value	SD	rawp	q value	R fold	name	Probe Set ID
1966	6.5	0.1776	1.75E-05	0.0381	51.404	202672_s_at	202672_s_at
2718	6.2	0.1182	2.63E-05	0.0381	33.809	203665_at	203665_at
11114	6.87	0.0288	8.77E-06	0.0381	32.063	36711_at	36711_at
1421	5.67	0.1569	4.39E-05	0.0381	29.246	202014_at	202014_at
5123	5.94	0.1183	3.51E-05	0.0381	28.912	209383_at	209383_at
11125	5.34	0.0453	5.26E-05	0.0381	15.798	37028_at	37028_at
3208	4.79	0.0485	6.14E-05	0.0381	12.023	204472_at	204472_at
2888	4.14	0.1084	7.89E-05	0.0381	10.127	203946_s_at	203946_s_at
1888	4.37	0.0592	7.02E-05	0.0381	10.005	202581_at	202581_at
213	4.01	0.0766	8.77E-05	0.0381	8.685	200664_s_at	200664_s_at
2817	3.37	0.2171	0.000114	0.0381	8.395	203810_at	203810_at
9446	3.86	0.0726	9.65E-05	0.0381	7.895	218723_s_at	218723_s_at
4983	3.16	0.1934	0.00014	0.0381	6.972	209189_at	209189_at
937	3.26	0.1	0.000123	0.0381	6.078	201466_s_at	201466_s_at
4275	3.1	0.1178	0.000149	0.0381	5.779	207714_s_at	207714_s_at
9356	3.47	0.0242	0.000105	0.0381	5.721	218611_at	218611_at
214	3.09	0.0994	0.000158	0.0381	5.563	200666_s_at	200666_s_at
1146	3.18	0.073	0.000132	0.0381	5.473	201694_s_at	201694_s_at
565	3.04	0.0969	0.000175	0.0381	5.347	201041_s_at	201041_s_at
3303	2.96	0.1077	0.000211	0.0381	5.232	204621_s_at	204621_s_at
2563	2.65	0.1518	0.000325	0.0381	4.739	203455_s_at	203455_s_at
4600	3.02	0.038	0.000184	0.0381	4.698	208744_x_at	208744_x_at
7556	2.9	0.0662	0.000228	0.0381	4.677	213988_s_at	213988_s_at
9959	2.53	0.1771	0.000404	0.0381	4.651	219475_at	219475_at
2759	2.82	0.0715	0.000254	0.0381	4.521	203725_at	203725_at

Table S5. *Cont.*

936	2.93	0.0389	0.000219	0.0381	4.493	201464_x_at	201464_x_at
2098	2.56	0.1385	0.000377	0.0381	4.406	202843_at	202843_at
3110	2.57	0.1322	0.000368	0.0381	4.376	204286_s_at	204286_s_at
341	2.84	0.0464	0.000246	0.0381	4.341	200800_s_at	200800_s_at
5684	2.66	0.087	0.000316	0.0381	4.266	210592_s_at	210592_s_at
3109	2.63	0.0768	0.000333	0.0381	4.119	204285_s_at	204285_s_at
7808	2.61	0.0732	0.000342	0.0381	4.045	214683_s_at	214683_s_at
4102	2.59	0.0651	0.00036	0.0381	3.942	206976_s_at	206976_s_at
11373	2.42	0.115	0.000474	0.0381	3.932	65588_at	65588_at
3 4083	2.05	0.2565	0.001079	0.0381	3.809	206907_at	206907_at
2149	2.22	0.1652	0.000693	0.0381	3.755	202912_at	202912_at
3734	2.18	0.1722	0.000763	0.0381	3.709	205543_at	205543_at
4 783	2.09	0.2171	0.001	0.0381	3.705	201289_at	201289_at
8420	2.39	0.0843	0.000518	0.0381	3.667	217144_at	217144_at
4637	2.49	0.0459	0.000439	0.0381	3.623	208786_s_at	208786_s_at
4259	2.44	0.0602	0.000465	0.0381	3.606	207630_s_at	207630_s_at
6782	2.33	0.0878	0.000605	0.0381	3.564	212723_at	212723_at
0 9516	2.14	0.1567	0.000877	0.0381	3.538	218810_at	218810_at
6781	2.36	0.0667	0.000561	0.0381	3.513	212722_s_at	212722_s_at
4114	2.23	0.1061	0.000684	0.0381	3.486	207030_s_at	207030_s_at
1535	2.4	0.0505	0.000482	0.0381	3.481	202147_s_at	202147_s_at
8257	2.15	0.1319	0.000842	0.0381	3.461	216248_s_at	216248_s_at
4842	2.39	0.0296	0.0005	0.0381	3.352	209020_at	209020_at
8 5608	1.98	0.1706	0.001211	0.0381	3.346	210357_s_at	210357_s_at
3304	2.2	0.0907	0.000728	0.0381	3.335	204622_x_at	204622_x_at
1534	2.2	0.0792	0.000719	0.0381	3.294	202146_at	202146_at
6 7815	2.08	0.116	0.001018	0.0381	3.235	214696_at	214696_at
10625	2.16	0.079	0.000816	0.0381	3.212	221577_x_at	221577_x_at
2097	2.2	0.0445	0.000737	0.0381	3.115	202842_s_at	202842_s_at
1 5654	2.1	0.0768	0.000974	0.0381	3.097	210512_s_at	210512_s_at
4364	2.16	0.0532	0.000807	0.0381	3.082	208078_s_at	208078_s_at
340	2.15	0.0504	0.000851	0.0381	3.058	200799_at	200799_at
4 9815	2	0.1007	0.001175	0.0381	3.035	219228_at	219228_at
0 10204	-1.83	0.1625	0.001579	0.0381	0.332	220035_at	220035_at
4 11028	-1.86	0.1635	0.001526	0.0381	0.332	222380_s_at	222380_s_at
7 3409	-1.86	0.169	0.001553	0.0381	0.331	204827_s_at	204827_s_at
3 2690	-2.13	0.0514	0.000904	0.0381	0.33	203628_at	203628_at
8 7271	-2.03	0.0919	0.001123	0.0381	0.329	213435_at	213435_at
2 1760	-1.97	0.1202	0.001246	0.0381	0.328	202421_at	202421_at
6 7078	-2.12	0.0596	0.00093	0.0381	0.328	213122_at	213122_at
1 3616	-1.91	0.1448	0.001412	0.0381	0.327	205258_at	205258_at
1 7148	-2.06	0.0817	0.001061	0.0381	0.327	213233_s_at	213233_s_at
10688	-2.19	0.0359	0.000754	0.0381	0.327	221688_s_at	221688_s_at
9 7972	-1.98	0.1182	0.001219	0.0381	0.326	215068_s_at	215068_s_at
0 9264	-1.92	0.143	0.001404	0.0381	0.326	218507_at	218507_at
1 93	-1.97	0.1248	0.001237	0.0381	0.324	200050_at	200050_at

Table S5. *Cont.*

5 2301	-2.09	0.0796	0.001009	0.0381	0.324	203123_s_at	203123_s_at
4 9971	-1.82	0.1982	0.001614	0.0381	0.324	219495_s_at	219495_s_at
8604	-2.2	0.0408	0.000702	0.0381	0.323	217779_s_at	217779_s_at
5 9431	-1.89	0.1724	0.001447	0.0381	0.323	218704_at	218704_at
3 5719	-1.83	0.205	0.001605	0.0381	0.322	210674_s_at	210674_s_at
5 2386	-1.86	0.1819	0.001535	0.0381	0.317	203232_s_at	203232_s_at
855	-2.18	0.0636	0.000772	0.0381	0.316	201368_at	201368_at
5 1736	-2.04	0.1154	0.001096	0.0381	0.314	202392_s_at	202392_s_at
6 10221	-1.99	0.1474	0.001193	0.0381	0.313	220085_at	220085_at
8833	-2.25	0.046	0.000658	0.0381	0.312	218019_s_at	218019_s_at
4 10012	-1.89	0.1933	0.001439	0.0381	0.31	219581_at	219581_at
2 2856	-2.01	0.1542	0.001158	0.0381	0.308	203888_at	203888_at
3 5204	-2	0.145	0.001167	0.0381	0.308	209494_s_at	209494_s_at
9747	-2.15	0.0878	0.000833	0.0381	0.308	219127_at	219127_at
4 6447	-2.12	0.1025	0.000912	0.0381	0.307	212298_at	212298_at
7930	-2.15	0.0979	0.00086	0.0381	0.306	214949_at	214949_at
6 9052	-1.86	0.2075	0.001544	0.0381	0.306	218259_at	218259_at
6297	-2.16	0.098	0.000798	0.0381	0.304	212126_at	212126_at
2522	-2.36	0.0308	0.000553	0.0381	0.302	203395_s_at	203395_s_at
7 7104	-1.75	0.2793	0.001877	0.0381	0.301	213153_at	213153_at
0 9990	-1.96	0.1974	0.001316	0.0381	0.301	219538_at	219538_at
3147	-2.16	0.1034	0.000789	0.0381	0.3	204352_at	204352_at
7 10827	-2.07	0.1363	0.001026	0.0381	0.3	221892_at	221892_at
2 9275	-2.06	0.152	0.00107	0.0381	0.299	218519_at	218519_at
0 3729	-2.1	0.1323	0.000965	0.0381	0.298	205534_at	205534_at
4116	-2.35	0.0448	0.00057	0.0381	0.297	207038_at	207038_at
5 2733	-2.12	0.1305	0.000921	0.0381	0.296	203685_at	203685_at
1 7075	-2.14	0.1244	0.000886	0.0381	0.296	213117_at	213117_at
10154	-2.16	0.119	0.000825	0.0381	0.296	219918_s_at	219918_s_at
3961	-2.18	0.1101	0.000781	0.0381	0.295	206373_at	206373_at
3 11010	-2.09	0.1493	0.000991	0.0381	0.294	222313_at	222313_at
3262	-2.25	0.0934	0.000667	0.0381	0.292	204554_at	204554_at
6395	-2.2	0.1103	0.000711	0.0381	0.291	212240_s_at	212240_s_at
7013	-2.39	0.0467	0.000509	0.0381	0.29	213032_at	213032_at
7247	-2.32	0.0752	0.000614	0.0381	0.288	213390_at	213390_at
8 6336	-2.07	0.1792	0.001035	0.0381	0.287	212170_at	212170_at
2948	-2.26	0.1029	0.00064	0.0381	0.284	204029_at	204029_at
9 9708	-2.07	0.1886	0.001044	0.0381	0.284	219069_at	219069_at
10922	-2.46	0.0392	0.000456	0.0381	0.283	222108_at	222108_at
7 6343	-1.96	0.2213	0.001289	0.0381	0.282	212179_at	212179_at
2 10811	-2.13	0.163	0.000895	0.0381	0.282	221869_at	221869_at
9873	-2.36	0.0806	0.000535	0.0381	0.279	219330_at	219330_at
3039	-2.19	0.1385	0.000746	0.0381	0.278	204172_at	204172_at

Table S5. *Cont.*

2 8503	-1.91	0.256	0.001421	0.0381	0.278	217523_at	217523_at
2855	-2.32	0.0998	0.000623	0.0381	0.277	203887_s_at	203887_s_at
7003	-2.34	0.0932	0.000596	0.0381	0.276	213019_at	213019_at
10285	-2.51	0.0442	0.000421	0.0381	0.274	220305_at	220305_at
9845	-2.35	0.0995	0.000579	0.0381	0.273	219283_at	219283_at
5271	-2.38	0.0933	0.000526	0.0381	0.27	209602_s_at	209602_s_at
8388	-2.34	0.1076	0.000588	0.0381	0.27	216971_s_at	216971_s_at
8 3461	-1.75	0.3515	0.001886	0.0381	0.269	204932_at	204932_at
8415	-2.46	0.0692	0.000447	0.0381	0.269	217122_s_at	217122_s_at
4930	-2.5	0.0643	0.00043	0.0381	0.267	209120_at	209120_at
3727	-2.39	0.0973	0.000491	0.0381	0.266	205527_s_at	205527_s_at
6986	-2.26	0.1488	0.000649	0.0381	0.266	212993_at	212993_at
3291	-2.55	0.0565	0.000386	0.0381	0.263	204602_at	204602_at
4110	-2.31	0.1432	0.000632	0.0381	0.261	207012_at	207012_at
6973	-2.54	0.0751	0.000395	0.0381	0.256	212977_at	212977_at
2 3927	-2.1	0.2497	0.000982	0.0381	0.254	206188_at	206188_at
10757	-2.36	0.1419	0.000544	0.0381	0.251	221786_at	221786_at
5249	-2.14	0.2355	0.000868	0.0381	0.25	209566_at	209566_at
3912	-2.7	0.045	0.000289	0.0381	0.248	206115_at	206115_at
1807	-2.66	0.0777	0.000307	0.0381	0.239	202478_at	202478_at
7581	-2.24	0.2699	0.000675	0.0381	0.229	214046_at	214046_at
11309	-2.66	0.1035	0.000298	0.0381	0.227	55872_at	55872_at
6404	-2.74	0.0869	0.000281	0.0381	0.224	212249_at	212249_at
7976	-2.79	0.0896	0.000263	0.0381	0.218	215073_s_at	215073_s_at
10406	-2.9	0.0994	0.000237	0.0381	0.201	220940_at	220940_at
3341	-3.08	0.0532	0.000167	0.0381	0.2	204686_at	204686_at
10304	-2.52	0.2415	0.000412	0.0381	0.197	220391_at	220391_at
1254	-2.59	0.2343	0.000351	0.0381	0.19	201818_at	201818_at
8676	-2.97	0.1117	0.000202	0.0381	0.188	217853_at	217853_at
6394	-2.78	0.1773	0.000272	0.0381	0.183	212239_at	212239_at
10380	-3	0.1291	0.000193	0.0381	0.178	220770_s_at	220770_s_at

Table S5. *Cont.*

Row	Gene Symbol	Gene Title	Pathway
1966	ATF3	activating transcription factor 3	Hypertrophy_model /// Hypertrophy_model /// Hypertrophy_model /// Hypertrophy_model /// Hypertrophy_model /// Smooth_muscle_contraction /// Smooth_muscle_contraction /// Smooth_muscle_contraction /// Smooth_muscle_contraction /// Smooth_muscle_contraction
2718	HMOX1	heme oxygenase (decycling) 1	---
11114	MAFF	v-maf musculoaponeurotic fibrosarcoma oncogene homolog F (avian)	Smooth_muscle_contraction
1421	PPP1R15A	protein phosphatase 1, regulatory (inhibitor) subunit 15A	---
5123	DDIT3	DNA-damage-inducible transcript 3	mRNA_processing_Reactome
11125	PPP1R15A	protein phosphatase 1, regulatory (inhibitor) subunit 15A	---
3208	GEM	GTP binding protein overexpressed in skeletal muscle	---
2888	ARG2	arginase, type II	---
1888	HSPA1B	heat shock 70 kDa protein 1B	---
213	DNAJB1	DnaJ (Hsp40) homolog, subfamily B, member 1	---
2817	DNAJB4	DnaJ (Hsp40) homolog, subfamily B, member 4	---
9446	C13orf15	chromosome 13 open reading frame 15	---
4983	FOS	v-fos FBJ murine osteosarcoma viral oncogene homolog	Smooth_muscle_contraction /// TGF_Beta_Signaling_Pathway
937	JUN	jun oncogene	Apoptosis /// Apoptosis_GenMAPP /// MAPK_Cascade /// Smooth_muscle_contraction /// TGF_Beta_Signaling_Pathway /// Wnt_signaling
4275	SERPINH1	serpin peptidase inhibitor, clade H (heat shock protein 47), member 1, (collagen binding protein 1)	---
9356	IER5	immediate early response 5	---
214	DNAJB1	DnaJ (Hsp40) homolog, subfamily B, member 1	---
1146	EGR1	early growth response 1	Ovarian_Infertility_Genes
565	DUSP1	dual specificity phosphatase 1	---
3303	NR4A2	nuclear receptor subfamily 4, group A, member 2	Nuclear_Receptors
2563	SAT1	spermidine/spermine N1-acetyltransferase 1	Nucleotide_Metabolism /// Nucleotide_Metabolism

Table S5. *Cont.*

4600	HSPH1	heat shock 105 kDa/110 kDa protein 1	---
7556	SAT1	spermidine/spermine N1-acetyltransferase 1	Nucleotide_Metabolism /// Nucleotide_Metabolism
9959	OSGIN1	oxidative stress induced growth inhibitor 1	---
2759	GADD45A	growth arrest and DNA-damage-inducible, alpha	Cell_cycle_KEGG /// G1_to_S_cell_cycle_Reactome
936	JUN	jun oncogene	Apoptosis /// Apoptosis_GenMAPP /// MAPK_Cascade /// Smooth_muscle_contraction /// TGF_Beta_Signaling_Pathway /// Wnt_signaling
2098	DNAJB9	DnaJ (Hsp40) homolog, subfamily B, member 9	---
3110	PMAIP1	phorbol-12-myristate-13-acetate-induced protein 1	---
341	HSPA1A /// HSPA1B	heat shock 70 kDa protein 1A /// heat shock 70 kDa protein 1B	---
5684	SAT1	spermidine/spermine N1-acetyltransferase 1	Nucleotide_Metabolism /// Nucleotide_Metabolism
3109	PMAIP1	phorbol-12-myristate-13-acetate-induced protein 1	---
7808	CLK1	CDC-like kinase 1	---
4102	HSPH1	heat shock 105 KDa/110 KDa protein 1	---
11373	LOC388796	hypothetical LOC388796	---
3 4083	TNFSF9	tumor necrosis factor (ligand) superfamily, member 9	---
2149	ADM	adrenomedullin	Smooth_muscle_contraction
3734	HSPA4L	heat shock 70 KDa protein 4-like	---
4 783	CYR61	cysteine-rich, angiogenic inducer, 61	Hypertrophy_model /// Hypertrophy_model /// Hypertrophy_model
8420	LOC648390 /// UBB	ubiquitin B /// similar to ubiquitin B precursor	DNA_replication_Reactome /// DNA_replication_Reactome /// Proteasome_Degradation /// Ribosomal_Proteins /// Ribosomal_Proteins
4637	MAP1LC3B	microtubule-associated protein 1 light chain 3 beta	---
4259	CREM	cAMP responsive element modulator	---
6782	JMJD6	jumonji domain containing 6	---
0 9516	ZC3H12A	zinc finger CCCH-type containing 12A	---
6781	JMJD6	jumonji domain containing 6	---
4114	CSRP2	cysteine and glycine-rich protein 2	---
1535	IFRD1	interferon-related developmental regulator 1	Hypertrophy_model
8257	NR4A2	nuclear receptor subfamily 4, group A, member 2	Nuclear_Receptors
4842	C20orf111	chromosome 20 open reading frame 111	---

Table S5. *Cont.*

8 5608	SMOX	spermine oxidase	---
3304	NR4A2	nuclear receptor subfamily 4, group A, member 2	Nuclear_Receptors
1534	IFRD1	interferon-related developmental regulator 1	Hypertrophy_model
6 7815	MGC14376	hypothetical protein MGC14376	---
10625	GDF15	growth differentiation factor 15	Cell_cycle_KEGG /// Cell_cycle_KEGG
2097	DNAJB9	DnaJ (Hsp40) homolog, subfamily B, member 9	---
1 5654	VEGFA	vascular endothelial growth factor A	Hypertrophy_model /// Hypertrophy_model
4364	SNF1LK	SNF1-like kinase	Hypertrophy_model
340	HSPA1A	heat shock 70 KDa protein 1A	---
4 9815	ZNF331	zinc finger protein 331	---
0 10204	NUP210	nucleoporin 210 KDa	---
4 11028	PDCD6	Programmed cell death 6	---
7 3409	CCNF	cyclin F	---
3 2690	IGF1R	insulin-like growth factor 1 receptor	---
8 7271	SATB2	SATB homeobox 2	---
2 1760	IGSF3	immunoglobulin superfamily, member 3	---
6 7078	TSPYL5	TSPY-like 5	---
1 3616	INHBB	inhibin, beta B	---
1 7148	KLHL9	kelch-like 9 (Drosophila)	---
10688	IMP3	IMP3, U3 small nucleolar ribonucleoprotein, homolog (yeast)	---
9 7972	FBXL18	F-box and leucine-rich repeat protein 18	---
0 9264	HIG2	hypoxia-inducible protein 2	---
1 93	ZNF146	zinc finger protein 146	---
5 2301	SLC11A2	solute carrier family 11 (proton-coupled divalent metal ion transporters), member 2	---
4 9971	ZNF180	zinc finger protein 180	---
8604	PNRC2	proline-rich nuclear receptor coactivator 2	---
5 9431	RNF43	ring finger protein 43	---
3 5719	PCDHA1 /// PCDHA10 /// PCDHA11 /// PCDHA12 /// PCDHA13 /// PCDHA2 /// PCDHA3 /// PCDHA4 /// PCDHA5 /// PCDHA6 /// PCDHA7 /// PCDHA8 /// PCDHA9 /// PCDHAC1 /// PCDHAC2	protocadherin alpha 9 /// protocadherin alpha subfamily C, 2 /// protocadherin alpha subfamily C, 1 /// protocadherin alpha 13 /// protocadherin alpha 12 /// protocadherin alpha 11 /// protocadherin alpha 10 /// protocadherin alpha 8 /// protocadherin alpha 7 /// protocadherin alpha 6 /// protocadherin alpha 5 /// protocadherin alpha 4 /// protocadherin alpha 3 /// protocadherin alpha 2 /// protocadherin alpha 1	---

Table S5. *Cont.*

5 2386	ATXN1	ataxin 1	---
855	ZFP36L2	zinc finger protein 36, C3H type-like 2	---
5 1736	PISD	phosphatidylserine decarboxylase	---
6 10221	HELLS	helicase, lymphoid-specific	Apoptosis /// Apoptosis
8833	PDXK	pyridoxal (pyridoxine, vitamin B6) kinase	---
4 10012	TSEN2	tRNA splicing endonuclease 2 homolog (S. cerevisiae)	---
2 2856	THBD	thrombomodulin	---
3 5204	PATZ1	POZ (BTB) and AT hook containing zinc finger 1	---
9747	ATAD4	ATPase family, AAA domain containing 4	---
4 6447	NRP1	neuropilin 1	---
7930	---	CDNA FLJ31919 fis, clone NT2RP7004964	---
6 9052	MKL2	MKL/myocardin-like 2	---
6297	---	CDNA clone IMAGE:4842353	---
2522	HES1	hairy and enhancer of split 1, (Drosophila)	---
7 7104	SETD1B	SET domain containing 1B	---
0 9990	WDR5B	WD repeat domain 5B	---
3147	TRAF5	TNF receptor-associated factor 5	---
7 10827	H6PD	hexose-6-phosphate dehydrogenase (glucose 1-dehydrogenase)	---
2 9275	SLC35A5	solute carrier family 35, member A5	---
0 3729	PCDH7	protocadherin 7	---
4116	SLC16A6	solute carrier family 16, member 6 (monocarboxylic acid transporter 7)	---
5 2733	BCL2	B-cell CLL/lymphoma 2	Apoptosis /// Apoptosis /// Apoptosis_GenMAPP /// Apoptosis_GenMAPP /// Apoptosis_KEGG /// Apoptosis_KEGG
1 7075	KLHL9	kelch-like 9 (Drosophila)	---
10154	ASPM	asp (abnormal spindle) homolog, microcephaly associated (Drosophila)	---
3961	ZIC1	Zic family member 1 (odd-paired homolog, Drosophila)	---
3 11010	---	Transcribed locus	---
3262	PPP1R3D	protein phosphatase 1, regulatory (inhibitor) subunit 3D	---
6395	PIK3R1	phosphoinositide-3-kinase, regulatory subunit 1 (p85 alpha)	---
7013	NFIB	nuclear factor I/B	---
7247	C19orf7	chromosome 19 open reading frame 7	---
8 6336	RBM12	RNA binding motif protein 12	---
2948	CELSR2	cadherin, EGF LAG seven-pass G-type receptor 2 (flamingo homolog, Drosophila)	GPCRDB_Other
9 9708	ANKRD49	ankyrin repeat domain 49	---
10922	AMIGO2	adhesion molecule with Ig-like domain 2	---

Table S5. *Cont.*

7 6343	SFRS18	splicing factor, arginine/serine-rich 18	---
2 10811	ZNF512B	zinc finger protein 512B	---
9873	VANGL1	vang-like 1 (van gogh, Drosophila)	---
3039	CPOX	coproporphyrinogen oxidase	Heme_Biosynthesis
2 8503	CD44	CD44 molecule (Indian blood group)	---
2855	THBD	thrombomodulin	---
7003	RANBP6	RAN binding protein 6	---
10285	MGC3260	hypothetical protein MGC3260	---
9845	C1GALT1C1	C1GALT1-specific chaperone 1	---
5271	GATA3	GATA binding protein 3	---
8388	PLEC1	plectin 1, intermediate filament binding protein 500 kDa	---
8 3461	TNFRSF11B	tumor necrosis factor receptor superfamily, member 11b (osteoprotegerin)	---
8415	LOC728661 /// SLC35E2	solute carrier family 35, member E2 /// similar to solute carrier family 35, member E2	---
4930	NR2F2	nuclear receptor subfamily 2, group F, member 2	Nuclear_Receptors
3727	GEMIN4	gem (nuclear organelle) associated protein 4	---
6986	---	MRNA; cDNA DKFZp667B1718 (from clone DKFZp667B1718)	---
3291	DKK1	dickkopf homolog 1 (Xenopus laevis)	---
4110	MMP16	matrix metallopeptidase 16 (membrane-inserted)	Matrix_Metalloproteinases
6973	CXCR7	chemokine (C-X-C motif) receptor 7	GPCRDB_Class_A_Rhodopsin-like /// Smooth_muscle_contraction
2 3927	---	---	---
10757	C6orf120	chromosome 6 open reading frame 120	---
5249	INSIG2	insulin induced gene 2	---
3912	EGR3	early growth response 3	---
1807	TRIB2	tribbles homolog 2 (Drosophila)	---
7581	FUT9	fucosyltransferase 9 (alpha (1,3) fucosyltransferase)	---
11309	ZNF512B	zinc finger protein 512B	---
6404	PIK3R1	phosphoinositide-3-kinase, regulatory subunit 1 (p85 alpha)	---
7976	NR2F2	nuclear receptor subfamily 2, group F, member 2	Nuclear_Receptors
10406	KIAA1641	KIAA1641	---
3341	IRS1	insulin receptor substrate 1	---
10304	ZBTB3	zinc finger and BTB domain containing 3	---
1254	AYTL2	acyltransferase like 2	---
8676	TNS3	tensin 3	---
6394	PIK3R1	phosphoinositide-3-kinase, regulatory subunit 1 (p85 alpha)	---
10380	LOC63920	transposon-derived Buster3 transposase-like	---

Table S6. HDAC1 assay.

HDAC Profiling Report for:			HDAC 1	
**Raw data**	**Conc. (M)**	**Compound 3**	**Trichostatin A**	**Conc. (M)**
	5.00E-05	1,093,635	−17,926	1.00E-05
	1.67E-05	1,037,070	−7744	3.33E-06
	5.56E-06	1,087,558	97,500	1.11E-06
	1.85E-06	1,076,952	49,599	3.70E-07
	6.17E-07	1,053,214	133,860	1.23E-07
	2.06E-07	1,043,907	334,102	4.12E-08
	6.86E-08	1,083,541	572,625	1.37E-08
	2.29E-08	1,023,350	753,577	4.57E-09
	7.62E-09	1,136,550	894,036	1.52E-09
	2.54E-09	1,027,389	947,204	5.08E-10
	DMSO	1,011,399	1,011,727	DMSO
**%Activity**	**Conc. (M)**	**Compound 3**	**Trichostatin A**	**Conc. (M)**
	5.00E-05	109.43	−1.79	1.00E-05
	1.67E-05	103.77	−0.77	3.33E-06
	5.56E-06	108.83	9.76	1.11E-06
	1.85E-06	107.77	4.96	3.70E-07
	6.17E-07	105.39	13.39	1.23E-07
	2.06E-07	104.46	33.43	4.12E-08
	6.86E-08	108.42	57.30	1.37E-08
	2.29E-08	102.40	75.41	4.57E-09
	7.62E-09	113.73	89.46	1.52E-09
	2.54E-09	102.81	94.78	5.08E-10
	DMSO	101.21	101.24	DMSO
	HILLSLOPE		−0.8597	
	IC50 (M)		1.78E-08	

Table S7. HDAC2 assay.

**HDAC Profiling Report for:**			**HDAC 2**	
**Raw data**				
	**Conc. (M)**	**Compound 3**	**Trichostatin A**	**Conc. (M)**
	5.00E-05	893,224	−24,522	1.00E-05
	1.67E-05	835,913	−13,755	3.33E-06
	5.56E-06	850,348	6115	1.11E-06
	1.85E-06	818,155	49,511	3.70E-07
	6.17E-07	810,526	183,597	1.23E-07
	2.06E-07	784,889	275,554	4.12E-08
	6.86E-08	741,056	472,297	1.37E-08
	2.29E-08	744,313	520,246	4.57E-09
	7.62E-09	693,681	631,849	1.52E-09
	2.54E-09	652,504	561,629	5.08E-10
	DMSO	666,520	8747	DMSO
**%Activity**				
	**Conc. (M)**	**Compound 3**	**Trichostatin A**	**Conc. (M)**
	5.00E-05	134.01	−3.68	1.00E-05
	1.67E-05	125.41	−2.06	3.33E-06
	5.56E-06	127.58	0.92	1.11E-06
	1.85E-06	122.75	7.43	3.70E-07
	6.17E-07	121.61	27.55	1.23E-07
	2.06E-07	117.76	41.34	4.12E-08
	6.86E-08	111.18	70.86	1.37E-08
	2.29E-08	111.67	78.05	4.57E-09
	7.62E-09	104.08	94.80	1.52E-09
	2.54E-09	97.90	84.26	5.08E-10
	DMSO	100.00	1.31	DMSO
	HILLSLOPE			
	IC50 (M)		-1.029	
			4.21E-08	

Table S8. HDAC3 assay.

**HDAC Profiling Report for:**		**HDAC 3**		
**Raw data**				
	**Conc. (M)**	**Compound 3**	**Trichostatin A**	**Conc. (M)**
	5.00E-05	683,472	−4031	1.00E-05
	1.67E-05	757,267	4630	3.33E-06
	5.56E-06	759,480	25,086	1.11E-06
	1.85E-06	794,280	60,861	3.70E-07
	6.17E-07	775,769	185,840	1.23E-07
	2.06E-07	771,409	324,648	4.12E-08
	6.86E-08	754,244	450,581	1.37E-08
	2.29E-08	736,505	717,207	4.57E-09
	7.62E-09	761,048	650005	1.52E-09
	2.54E-09	722,321	676,532	5.08E-10
	DMSO	661,081	705,108	DMSO
**%Activity**				
	**Conc. (M)**	**Compound 3**	**Trichostatin A**	**Conc. (M)**
	5.00E-05	101.14	−0.60	1.00E-05
	1.67E-05	112.06	0.69	3.33E-06
	5.56E-06	112.39	3.71	1.11E-06
	1.85E-06	117.54	9.01	3.70E-07
	6.17E-07	114.80	27.50	1.23E-07
	2.06E-07	114.16	48.04	4.12E-08
	6.86E-08	111.62	66.68	1.37E-08
	2.29E-08	108.99	106.14	4.57E-09
	7.62E-09	112.62	96.19	1.52E-09
	2.54E-09	106.89	100.12	5.08E-10
	DMSO	97.83	104.34	DMSO
	HILLSLOPE		−0.9811	
	IC50 (M)		3.56E-08	

Table S9. HDAC8 assay.

**HDAC Profiling Report for:**			**HDAC 8**	
**Raw data**				
	**Conc. (M)**	**Compound 3**	**Trichostatin A**	**Conc. (M)**
	5.00E-05	988,812	10,430	1.00E-05
	1.67E-05	1,240,425	191,730	3.33E-06
	5.56E-06	1,153,650	413,811	1.11E-06
	1.85E-06	1,131,422	582,860	3.70E-07
	6.17E-07	1,142,163	264,081	1.23E-07
	2.06E-07	1,205,483	1,105,830	4.12E-08
	6.86E-08	1,136,825	1,182,397	1.37E-08
	2.29E-08	1,031,471	1,052,439	4.57E-09
	7.62E-09	1,003,152	1,111,795	1.52E-09
	2.54E-09	908,412	1,019,259	5.08E-10
	DMSO	952,572	1,046,309	DMSO
**%Activity**				
	**Conc. (M)**	**Compound 3**	**Trichostatin A**	**Conc. (M)**
	5.00E-05	94.88	1.00	1.00E-05
	1.67E-05	119.03	18.40	3.33E-06
	5.56E-06	110.70	39.71	1.11E-06
	1.85E-06	108.57	55.93	3.70E-07
	6.17E-07	109.60	25.34	1.23E-07
	2.06E-07	115.68	106.11	4.12E-08
	6.86E-08	109.09	113.46	1.37E-08
	2.29E-08	98.98	100.99	4.57E-09
	7.62E-09	96.26	106.69	1.52E-09
	2.54E-09	87.17	97.81	5.08E-10
	DMSO	91.41	100.40	DMSO
	HILLSLOPE		−1.036	
	IC50 (M)		5.67E-07	

Table S10. HDAC4 assay.

HDAC Profiling Report for:		HDAC 4		
**Raw data**				
	**Conc. (M)**	**Compound 3**	**Trichostatin A**	**Conc. (M)**
	1.00E-04	5,993,680.375	3,008,268.375	1.00E-05
	3.33E-05	5,808,967.375	5,198,297.375	3.33E-06
	1.11E-05	7,405,389.375	7,509,677.375	1.11E-06
	3.70E-06	7,094,447.375	8,041,934.375	3.70E-07
	1.23E-06	5,616,796.375	8,129,562.375	1.23E-07
	4.12E-07	5,790,174.375	8,331,624.375	4.12E-08
	1.37E-07	5,649,893.375	8,034,621.375	1.37E-08
	4.57E-08	6,507,401.375	8,665,961.375	4.57E-09
	1.52E-08	6,887,892.375	8,879,331.375	1.52E-09
	5.08E-09	6,058,946.375	8,636,970.375	5.08E-10
	DMSO	6,282,617.375	8,621,051.375	DMSO
**%Activity**				
	**Conc. (M)**	**Compound 3**	**Trichostatin A**	**Conc. (M)**
	1.00E-04	77.17	38.73	1.00E-05
	3.33E-05	74.79	66.93	3.33E-06
	1.11E-05	95.34	96.69	1.11E-06
	3.70E-06	91.34	103.54	3.70E-07
	1.23E-06	72.32	104.67	1.23E-07
	4.12E-07	74.55	107.27	4.12E-08
	1.37E-07	72.74	103.45	1.37E-08
	4.57E-08	83.78	111.57	4.57E-09
	1.52E-08	88.68	114.32	1.52E-09
	5.08E-09	78.01	111.20	5.08E-10
	DMSO	80.89	111.00	DMSO
	HILLSLOPE		1.076	
	IC50 (M)		5.52E-06	

Table S11. HDAC5 assay.

**HDAC Profiling Report for:**			**HDAC 5**	
**Raw data**				
	**Conc. (M)**	**Compound 3**	**TMP269**	**Conc. (M)**
	1.00E-04	3,558,500.667	133,462.6667	1.00E-05
	3.33E-05	5,036,154.667	222,926.6667	3.33E-06
	1.11E-05	4,125,271.667	773,759.6667	1.11E-06
	3.70E-06	5,591,254.667	2,013,947.667	3.70E-07
	1.23E-06	4,367,792.667	3,323,326.667	1.23E-07
	4.12E-07	5,637,649.667	4,148,331.667	4.12E-08
	1.37E-07	4,699,396.667	4,597,377.667	1.37E-08
	4.57E-08	4,825,308.667	4,820,119.667	4.57E-09
	1.52E-08	4,708,472.667	4,759,513.667	1.52E-09
	5.08E-09	3,774,142.667	5,063,291.667	5.08E-10
	DMSO	4,048,569.667	4,987,192.667	DMSO
**%Activity**				
	**Conc. (M)**	**Compound 3**	**TMP269**	**Conc. (M)**
	1.00E-04	78.76	2.95	1.00E-05
	3.33E-05	111.47	4.93	3.33E-06
	1.11E-05	91.31	17.13	1.11E-06
	3.70E-06	123.76	44.58	3.70E-07
	1.23E-06	96.68	73.56	1.23E-07
	4.12E-07	124.79	91.82	4.12E-08
	1.37E-07	104.02	101.76	1.37E-08
	4.57E-08	106.80	106.69	4.57E-09
	1.52E-08	104.22	105.35	1.52E-09
	5.08E-09	83.54	112.07	5.08E-10
	DMSO	89.61	110.39	DMSO
	HILLSLOPE		−1.023	
	IC50 (M)		2.42E-07	

Table S12. HDAC7 assay.

**HDAC Profiling Report for:**			**HDAC 7**	
**Raw data**				
	**Conc. (M)**	**Compound 3**	**TMP269**	**Conc. (M)**
	1.00E-04	7,823,263.4	58,766.4	1.00E-05
	3.33E-05	9,155,499.4	231,645.4	3.33E-06
	1.11E-05	8,809,533.4	523,432.4	1.11E-06
	3.70E-06	9,047,156.4	1,965,361.4	3.70E-07
	1.23E-06	8,424,306.4	4,080,044.4	1.23E-07
	4.12E-07	8,488,379.4	704,0639.4	4.12E-08
	1.37E-07	7,171,119.4	8,526,019.4	1.37E-08
	4.57E-08	8,594,533.4	8,899,709.4	4.57E-09
	1.52E-08	6817134.4	8,964,461.4	1.52E-09
	5.08E-09	7840947.4	9,600,795.4	5.08E-10
	DMSO	7252110.4	10,084,442.4	DMSO
**%Activity**				
	**Conc. (M)**	**Compound 3**	**TMP269**	**Conc. (M)**
	1.00E-04	90.25	0.68	1.00E-05
	3.33E-05	105.62	2.67	3.33E-06
	1.11E-05	101.63	6.04	1.11E-06
	3.70E-06	104.37	22.67	3.70E-07
	1.23E-06	97.19	47.07	1.23E-07
	4.12E-07	97.92	81.22	4.12E-08
	1.37E-07	82.73	98.36	1.37E-08
	4.57E-08	99.15	102.67	4.57E-09
	1.52E-08	78.64	103.42	1.52E-09
	5.08E-09	90.46	110.76	5.08E-10
	DMSO	83.66	116.34	DMSO
	HILLSLOPE		−1.063	
	IC50 (M)		9.85E-08	

Table S13. HDAC9 assay.

**HDAC Profiling Report for:**			**HDAC 9**	
**Raw data**				
	**Conc. (M)**	**Compound 3**	**TMP269**	**Conc. (M)**
	1.00E-04	6,280,796.2	−3791.8	1.00E-05
	3.33E-05	6,861,699.2	41,121.2	3.33E-06
	1.11E-05	6,201,048.2	208,251.2	1.11E-06
	3.70E-06	6,364,650.2	407,051.2	3.70E-07
	1.23E-06	6,903,225.2	1,034,443.2	1.23E-07
	4.12E-07	6,286,176.2	2,909,830.2	4.12E-08
	1.37E-07	5,235,179.2	4,847,731.2	1.37E-08
	4.57E-08	6,575,210.2	6,426,035.2	4.57E-09
	1.52E-08	5675121.2	7,301,122.2	1.52E-09
	5.08E-09	5716508.2	7,455,858.2	5.08E-10
	DMSO	6378242.2	7,682,762.2	DMSO
**%Activity**				
	**Conc. (M)**	**Compound 3**	**TMP269**	**Conc. (M)**
	1.00E-04	89.34	−0.05	1.00E-05
	3.33E-05	97.60	0.58	3.33E-06
	1.11E-05	88.20	2.96	1.11E-06
	3.70E-06	90.53	5.79	3.70E-07
	1.23E-06	98.19	14.71	1.23E-07
	4.12E-07	89.41	41.39	4.12E-08
	1.37E-07	74.46	68.95	1.37E-08
	4.57E-08	93.52	91.40	4.57E-09
	1.52E-08	80.72	103.85	1.52E-09
	5.08E-09	81.31	106.05	5.08E-10
	DMSO	90.72	109.28	DMSO
	HILLSLOPE		−1.036	
	IC50 (M)		2.38E-08	

Table S14. HDAC6 assay.

HDAC Profiling Report for:			HDAC 6	
**Raw data**	**Conc. (M)**	**Compound 3**	**Trichostatin A**	**Conc. (M)**
	1.20E-04	1,106,550.917	−54,261.08333	1.00E-05
	4.00E-05	1,825,036.917	−11,767.08333	3.33E-06
	1.33E-05	2,550,355.917	−58,888.08333	1.11E-06
	4.44E-06	2,762,459.917	11,374.91667	3.70E-07
	1.48E-06	2,709,307.917	−6533.083333	1.23E-07
	4.94E-07	2,588,473.917	145,032.9167	4.12E-08
	1.65E-07	2,600,774.917	255,164.9167	1.37E-08
	5.49E-08	2,838,774.917	753,719.9167	4.57E-09
	1.83E-08	2,964,065.917	1,251,580.917	1.52E-09
	6.10E-09	2,681,842.917	2,343,261.917	5.08E-10
	DMSO	2,756,697.917	3,059,961.917	DMSO
**%Activity**	**Conc. (M)**	**Compound 3**	**Trichostatin A**	**Conc. (M)**
	1.20E-04	38.05	−1.87	1.00E-05
	4.00E-05	62.75	−0.40	3.33E-06
	1.33E-05	87.69	−2.02	1.11E-06
	4.44E-06	94.98	0.39	3.70E-07
	1.48E-06	93.16	−0.22	1.23E-07
	4.94E-07	89.00	4.99	4.12E-08
	1.65E-07	89.43	8.77	1.37E-08
	5.49E-08	97.61	25.92	4.57E-09
	1.83E-08	101.92	43.03	1.52E-09
	6.10E-09	92.21	80.57	5.08E-10
	DMSO	94.79	105.21	DMSO
	HILLSLOPE	−1.19	−1.036	
	IC50 (M)	8.09E-05	1.29E-09	

Figure S16. Structure of diterpene analogue.

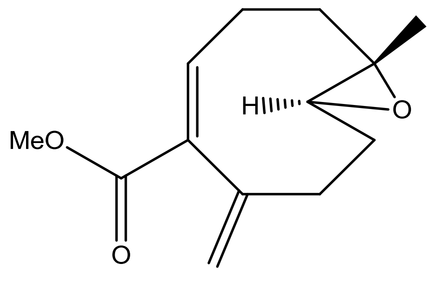

Molecular Weight: 222.28

© 2014 by the authors; licensee MDPI, Basel, Switzerland. This article is an open access article distributed under the terms and conditions of the Creative Commons Attribution license (http://creativecommons.org/licenses/by/3.0/).
